# Prognostic model revealing pyroptosis-related signatures in oral squamous cell carcinoma based on bioinformatics analysis

**DOI:** 10.1038/s41598-024-56694-y

**Published:** 2024-03-14

**Authors:** Lu Qi, Zhangui Tang

**Affiliations:** Hunan Key Laboratory of Oral Health Research, Hunan Clinical Research Center of Oral Major Diseases and Oral Health, Xiangya Stomatological Hospital, Xiangya School of Stomatology, Central South University, Changsha, 410000 China

**Keywords:** Pyroptosis, Gene enrichment analysis, Immune infiltration, Prognosis, Oral squamous cell carcinoma, Biochemistry, Biological techniques, Cancer, Computational biology and bioinformatics

## Abstract

One of the most common oral carcinomas is oral squamous cell carcinoma (OSCC), bringing a heavy burden to global health. Although progresses have been made in the intervention of OSCC, 5 years survival of patients suffering from OSCC is poor like before regarding to the high invasiveness of OSCC, which causes metastasis and recurrence of the tumor. The relationship between pyroptosis and OSCC remains to be further investigated as pyroptosis in carcinomas has gained much attention. Herein, the key pyroptosis-related genes were identified according to The Cancer Genome Atlas (TCGA) dataset. Additionally, a prognostic model was constructed based upon three key genes (CTLA4, CD5, and IL12RB2) through least absolute shrinkage and selection operator (LASSO) analyses, as well as univariate and multivariate COX regression in OSCC. It was discovered that the high expression of these three genes was associated with the low-risk group. We also identified *LAIR2* as a hub gene, whose expression negatively correlated with the risk score and the different immune cell infiltration. Finally, we proved that these three genes were independent prognostic factors linked to overall survival (OS), and reliable consequences could be predicted by this model. Our study revealed the relationship between pyroptosis and OSCC, providing insights into new treatment targets for preventing and treating OSCC.

## Introduction

One of the most frequent malignancies is head and neck squamous cell carcinoma (HNSC) mainly originating in the mouth, pharynx, and throat mucosas, of which oral squamous cell carcinoma (OSCC), laryngeal squamous cell carcinoma (LSCC), and nasopharyngeal carcinoma are the major subtypes^1^. Among them, OSCC becomes the most common subtype, reaching more than 80% of oral malignancies worldwide^2,3^. The occurrence and development of OSCC is associated with the accumulation of genome mutations, and the risk factors including tobacco, alcohol, and human papillomavirus (HPV)^4^. Technical progresses have been made in these years for treating OSCC, but the 5-years post-diagnosis survival still remains unsatisfactory considering the high invasiveness of OSCC^5^. Therefore, efforts should be made to develop novel strategies for the treatment and prevention of OSCC. Identifying the potential molecular biomarkers for early diagnostics may be helpful to develop novel diagnostic and therapeutic approaches for OSCC.

The programmed cell death is mainly divided into pyroptosis and apoptosis. Pyroptosis is a well-known inflammatory form, which is resulted from inflammasomes, and causes cell lysis by gasdermin D cleavage and the activation of pro-inflammatory cytokines, including IL-1β and IL-18^6^. Apoptosis is thought to be a basic mechanism of anti-tumor responses^7,8^, yet the relationship between pyroptosis and tumorigenesis remains elusive. Of late, pyroptosis has been a new frontier in cancer researches, which is capable of regulating tumor cell proliferation, invasion and metastasis^9^. Pyroptosis was reported to correlate with multiple categories of carcinomas^10^, implying the guiding significance of pyroptosis-related molecules and pathways in the prevention and treatment of different cancers. In recent years, the advancement of interaction prediction methods in computational biology has provided insights for identifying genetic markers in certain diseases^11,12^. It was reported that a pyroptosis-related gene signature was prognostic for HNSC patient^13^. Several recent studies performed bioinformatics analyses and found that the pyroptosis-related signature was prognostic for the level of immune cell infiltration benefit to immunotherapies in OSCC^14–16^. Other evidences were also provided about the role of pyroptosis in the development and treatment of OSCC^17,18^. However, the precise relationship between pyroptosis and OSCC is still elusive which needs to be further investigated.

Herein, we identified key pyroptosis-related genes in OSCC according to TCGA dataset and performed gene enrichment analysis based upon the genes. A prognostic model was constructed based upon three key genes (CTLA4, CD5, and IL12RB2) in OSCC through the aforementioned approaches. Furthermore, it was found that the high expression of these three gene was linked to the low-risk group. The leukocyte-associated Ig-like receptor-2 (LAIR2) was also identified as a hub gene, whose expression negatively correlated with the risk score and linked with the different immune cell infiltration. Ultimately, we examined the relation between prognosis and clinical factors to explore the function of pyroptosis for the clinical treatment of OSCC.

## Methods

### Data download

Download gene expression data (FPKM and counts) of TCGA HNSC RNA sequencing data (n = 546), somatic mutation data (MuTect2 version; n = 508), and masked copy number segment data (n = 524) from UCSC xena (http://xena.ucsc.edu/)^19^. The SNP6 GRCh38 Remapped Probeset File was downloaded for Copy Number Variation Analysis data from the TCGA Genomic Data Commons (GDC) database (https://portal.gdc.cancer.gov) as reference information on masked copy number segment data, which are used for the analysis of Genomic Identification of Significant Targets in Cancer (GISTIC 2.0). Download the corresponding clinical pathological features and prognosis information of patients suffering from OSCC, including gender, age, stage, etc. Samples with the primary site as “Lip”, “Palate”, “Gum”, “Base of tongue”, “Floor of mouth”, “Other and unclear sites in lip, oral cavity and pharynx”, “Other and unclear sits of tongue”, and “Other and unspecified parts of mouth” are selected for OSCC, which includes 32 normal samples and 346 cancer samples. Download OSCC sequencing data GSE41613 and GSE111390 from GEO database (https://www.ncbi.nlm.nih.gov/geo). The GSE41613 platform is GPL570, containing a total of 97 OSCC samples without any paracancerous or normal samples. The platform of GSE111390 is GPL6480, containing 33 samples of oropharyngeal cancer. 14 samples with topology attributes of “base of tongue” and “soft pad” are selected as OSCC samples, without any paracancerous or normal samples. The sample information of TCGA OSCC and validation dataset is shown in Table S1. Download 53 human cell pyroptosis-related genes from references PMID33828074^20^, PMID34226539^21^, PMID34179006^22^, and PMID35364268^23^, as shown in Table S2.

### GSVA analysis

Gene Set Variation Analysis (GSVA) is a non-parametric unsupervised analysis approach which evaluates if various metabolic pathways are plentiful in disparate specimens, with the gene expression matrix into the expression matrix of gene sets among different specimens^24^. To investigate biological processes associated with OSCC, we calculated the cell pyroptosis score (gsva score) of each sample by means of R package GSVA (v1.40.1)^24^, on the basis of the gene expression profile dataset and 33 pyroptosis genes. Subsequently, we used the R package surv_Cutpoint() and survey_Categorize() of survminer to find the optimal cutoff as the threshold, and the specimens were split into high pyroptosis scoring group and low pyroptosis scoring group. This was achieved by quickly obtaining the optimal survival group through best separation, and conducting Kaplan Meier (KM) survival analysis based upon OS time, in order to detect the survival distinctions between high and low scoring groups.

### Differentially expressed genes analysis

With a view to revealing the distinction of gene expression between OSCC high and low pyroptosis scoring groups, differentially expressed gene (DEG) analysis was conducted. R packet limma was employed, for the purpose of performing the DEG analysis on TCGA OSCC data^25^, with |log2 fold change| (|log2FC|) >  = 1 and adjust *P* value < 0.05 set as the threshold for DEGs. With log2FC > 1 and adjust *P* value < 0.05, genes were concerned as upregulated DEGs, while with log2FC < − 1 and adjust *P* value < 0.05 genes were as downregulated DEGs. Subsequently, volcano plots and heat maps were applied to visualize significant DEGs related to pyroptosis scores. The volcanic plot was described by means of R package ggplot2 (v3.3.5, https://ggplot2.tidyverse.org/). The heat map was described by means of R package pheatmap (v1.0.12).

### Correlation network analysis

Weighted correlation network analysis (WGCNA) is an integrative biology way of depicting the gene association modes in disparate specimens, which is capable of being adopted so that the highly synergistic gene sets were identified, with gene sets associated with phenotypic traits, and treatment targets or candidate biomarkers based on the gene set interconnectedness as well as the correlation between phenotypes and gene sets^26^. MAD TOP15000 genes were analyzed for gene co-expression identification to identify modules related to phenotypic traits using R package WGCNA (v1.70.3)^26^. A network of scale-free topology was constructed through selecting the soft thresholds using the pick Soft Threshold function (scale free R^2^ = 0.9). The matrix of adjacency was converted into a topological overlap matrix (TOM), genes were split into disparate gene modules based upon TOM dissimilarity measures, and merge Cut Height was set as 0.25 and min Module Size as 10 to recognize the key modules. The modules of high correlation with the pyroptosis gsva score (|correlation coefficient|> = 0.5 and *P* value < 0.05 were chosen.

### Identification of key genes in OSCC

To further identify key genes related to both OSCC and cell pyroptosis, we intersected differentially expressed pyroptosis-related genes (DEPGs) with module genes associated with pyroptosis (gsva score) recognized by WGCNA. These intersected genes are considered to be key genes in OSCC, which are related to both OSCC occurrence and pyroptosis. Subsequently, we constructed a protein–protein interaction (PPI) network (high confidence 0.9) based on the search tool for the retrieval of interaction gene/proteins (STRING) database of these key genes^27^, and visualized the PPI network using Cytoscape (v3.8.2) software^28^. The McCreight (MCC) algorithm using the cytoHubba plugin (v0.1) was used to obtain hub nodes^29^, and genes with TOP5 MCC scores were used as the hub genes. The Pearson correlation among the hub gene expression levels was calculated, which was deeply visualized by heat maps using R packet coreplot (v0.92).

### Protein–protein interaction (PPI)

There is strong correlation among the gene expressions, particularly those regulating the identical biological course. Thereby, in order to uncover the association among DEGs linked to pyroptosis, a PPI network was set up. By means of STRING (https://www.string-db.org) database^27^, we took the aforementioned genes as input, with a confidence threshold of 0.9, and constructed a PPI network which was visualized by Cytoscape (v3.8.2) software^28^. Using cytoHubba (v0.1)^29^, we mined hub nodes based on the MCC algorithm, with genes of the TOP5 MCC score as hubs genes. The Pearson correlation among the hub gene expression levels was also calculated, which was visualized by heat maps using R packet coreplot (v0.92).

### Unsupervised clustering analysis

Non-negative Matrix Factorization (NMF) unsupervised clustering analysis was performed on dataset samples based on key genes related to OSCC and pyroptosis, using the NMF package (v0.23.0) of R^30^. Finally, principal component analysis (PCA) and visualization of the samples were performed based on NMF classification and gene expression features, using the FactoMineR package (v2.4)^31^, as well as the factoextra package (v1.0.7, https://rdocumentation.org/packages/factoextra/versions/1.0.7). Subsequently, we conducted KM survival analysis of different NMF categories.

### Construction and evaluation of prognostic models

On account of the correlation between pyroptosis and OSCC, it is exceedingly potential to set up a diagnostic model on the basis of DEGs linked to pyroptosis. The univariate regression analysis was first implemented, with the aim of screening for key genes in OSCC (*P* value < 0.05), and then performed LASSO regression analysis for further screening. We implemented this method using R package glmnet (v4.1.2, https://glmnet.stanford.edu) and selected the optimal lambda value. With regression analysis done, merely genes with coefficients except 0 were kept as genes for constructing prognostic models. Subsequently, the association between risk score and pyroptosis score (gsva score) was computed, with a view to validating the predicted efficiency of the diagnostic model. At the same time, we used the survey_Cutpoint() and survey_Categorize () of surveyminer in R package to find the optimal cutoff as the threshold, and split the specimens into high-risk and low-risk groups. This was achieved by quickly obtaining the optimal survival group through best separation, and conducting the survival analysis of KM based upon OS (overall survival) time, so that to explore the survival distinctions between those two groups. Then, the area under the curve (AUC) of the receiver-operating curve (ROC) at 1, 3, and 10 years was further evaluated using R package pROC (v1.18.0)^32^. In order to detect the impact of prognostic genes related to pyroptosis on survival, we conducted the analysis on risk factors, with the objective to detecting the association between survival rate and gene expression levels. To further demonstrate the predictive robustness of the model, dataset GSE41613 and GSE111390 were used for validation. The ROC was also described; besides, the AUC was figured out to assess the predicted efficiency of the model.

### Gene enrichment analysis

The same differential analysis method was used to perform differential expression analysis on high-risk and low-risk groups (see Gene Differential Expression Analysis section). And the enrichment analysis was implemented. Based upon DEGs in order to explore the biological functions involved. Gene Ontology (GO) enrichment analysis becomes an approach in common use for large-scale functional enrichment researches on genes at disparate dimensions, which is ordinarily conducted at three levels: biological process (BP), molecular function (MF), and cellular component (CC)^33^. Kyoto Encyclopedia of Genes and Genomes (KEGG) is an extensively applied database which preserves the information on genomes, diseases, biological pathways, and drugs^34^. Using R package clusterProfiler (v4.0.5)^35^, GO functional annotation and KEGG pathway enrichment analysis were implemented on all significant DEGs related to pyroptosis so as to recognize markedly plentiful BPs and pathways. The enrichment consequences were further known by means of R package GOplot (v1.0.2)^36^ and enrichplot (v1.12.3, https://yulab-smu.top/biomedical-knowledge-mining-book/), besides, the significance thresholds for enrichment analysis were designated as *P* < 0.05. Gene Set Enrichment Analysis (GSEA) is one computational approach that can be employed to determine if the predefined genes exhibit statistical distinctions between two biological forms, and is typically adopted, with the aim of analyzing the alterations in pathway and biological activities among dataset samples^37^. It evaluates the distributive tendency of genes in the genes ranked by phenotypic correlation using a pre-defined gene set to determine their contribution to the specific phenotype. In order to evaluate the variations of BPs between those groups of patients, the reference gene set “c2. cp. kegg. v7.4 Entrez. gmt” was downloaded from the MSigDB database (https://www.gsea-msigdb.org/gsea/msigdb/)^38^. Based upon the gene expression profile dataset, we used the GSEA function of R package clusterProfiler (v4.0.5) to fulfill enrichment analysis and dataset visualization^35^. *P* value < 0.05 was regarded to be significant statistically.

### Construction of mRNA-miNRA interaction network

The mRNA-miRNA regulatory network involves different RNA molecules, including mRNA, miRNA, etc., which provides us with a new perspective for transcriptomic researches and helps to comprehensively and deeply explain some biological phenomena. To analyze the association between key genes of OSCC and miRNAs at the post-transcriptional level, we obtained the interaction pairs of key genes and miRNAs related to both OSCC and pyroptosis from the Tarbase database^39^ and miRDB database^40^. Both databases assumed that they had higher accuracy. Venn diagram was drawn using ggvenn (v0.1.9).

### Immune infiltration analysis

As a loaded comprehensive system, the immune microenvironment chiefly consisted of normal fibroblasts, immune cells, interstitial tissues, inflammatory cells, as well as diverse cytokines and chemokines. The infiltration analysis on immune cells plays a crucial essential part in guiding researches about diseases and treatment prediction. With a view to deeply analyzing the association between key genes related to OSCC and pyroptosis, as well as the levels of immune cell infiltration, CIBERSORT was adopted to evaluate the immune cell infiltration level, based upon the LM22 background gene set from the CIBERSORT website (https://cibersort.stanford.edu/)^41^, and the content of 22 categories of each patient’s immune cells was calculated, with the objective to illuminating the level of the infiltration. Those results were presented by box plots using R package ggplot2 (v3.3.5). Immune cells with prominent differences between patients suffering from high and low risks of pyroptosis scores may have a closer relationship with OSCC key genes. R package ggExtra (v0.9, https://github.com/daattali/ggExtra) was used to draw scatter plots of prominent differences in immune cell infiltration levels and OSCC key gene expression values with *P* value < 0.05, additionally, the relationship curve was then plotted.

### Mutation signatures analysis

Considering that high-risk and low-risk groups may have disparate roles during the tumor development, we explored the differences of the mutation signatures of the high-risk group. Based upon OSCC, the somatic mutation data was presented using the Maftools package (version 2.8.5), for the purpose of displaying a panoramic view of OSCC mutations^42^. Based on the copy number change data and relevant annotation data concerning carcinoma samples, GISTIC_2.0 tool module (v2.0.23, default parameter) in GenePattern website (https://cloud.genepattern.org/gp/pages/index.jsf)^43^ was employed so as to analyze region information of significant amplification and deletion in primary squamous cell carcinoma (PSCC) samples, and GRCh38 was used as the reference genome. Then, Maftools was used for visualization. In addition, to find the distinctions in mutation signatures between these groups, the differences of mutation signatures were figured out using R package deconstructSigs (v1.8.0)^44^, using signatures. anture2013 as the reference data. The Tumor Immune Dysfunction and Exclusion (TIDE) score represents the sensitiveness to immune checkpoints^45,46^, additionally, the TIDE score of each tumor specimen can serve as an alternative biomarker predicting the response to blockade of the immune checkpoint. The TIDE score of lung adenocarcinoma and the differences between high-risk and low-risk groups were calculated on the TIDE website (https://tide.dfci.harvard.edu/login/) on the basis of the Predict Response module.

### Clinical factor analysis

A prognostic model was set up by means of gene expression data from OSCC patients with clinical and mutation information. Based upon the gender, age, clinical phase, and tumor phase of OSCC patients, univariate COX analysis and multivariate COX analysis were conducted, in order to calculate the independent predictive ability of clinical pathological characteristic on OS. The related indicators were included in the model, besides, a clinical prediction nomogram and corresponding Calibrate correction chart were constructed. The predictive effectiveness of the model was further evaluated.

### Statistical analysis

Apart from the total data calculations, the statistical analysis was conducted in R language (v4.1.0). Benjamin Hochberg (BH) test was applied to the multiple test corrections. Besides, false discovery rate (FDR) correction was applied to multiple tests, with the aim of reducing false positive rates. With a view to comparing two consecutive variables, Mann Whitney U test (i.e. Wilcoxon rank-sum test) was used to analyze the distinctions between non-normal distribution variables. R survival package (v3.2.11) was adopted to analyze the survival^47^, and the survival curve of KM was plotted with the aim of displaying the survival differences. The test of log-rank was applied, in order to assess the significance of the survival distinctions between two patient groups. Univariate and multivariate COX analysis were performed to measure the independent prognostic factors. The total statistical *P* values were bilateral. *P* value < 0.05 were thought to be significant statistically.

## Result

### Identification of key genes in OSCC

**Figure 1:**
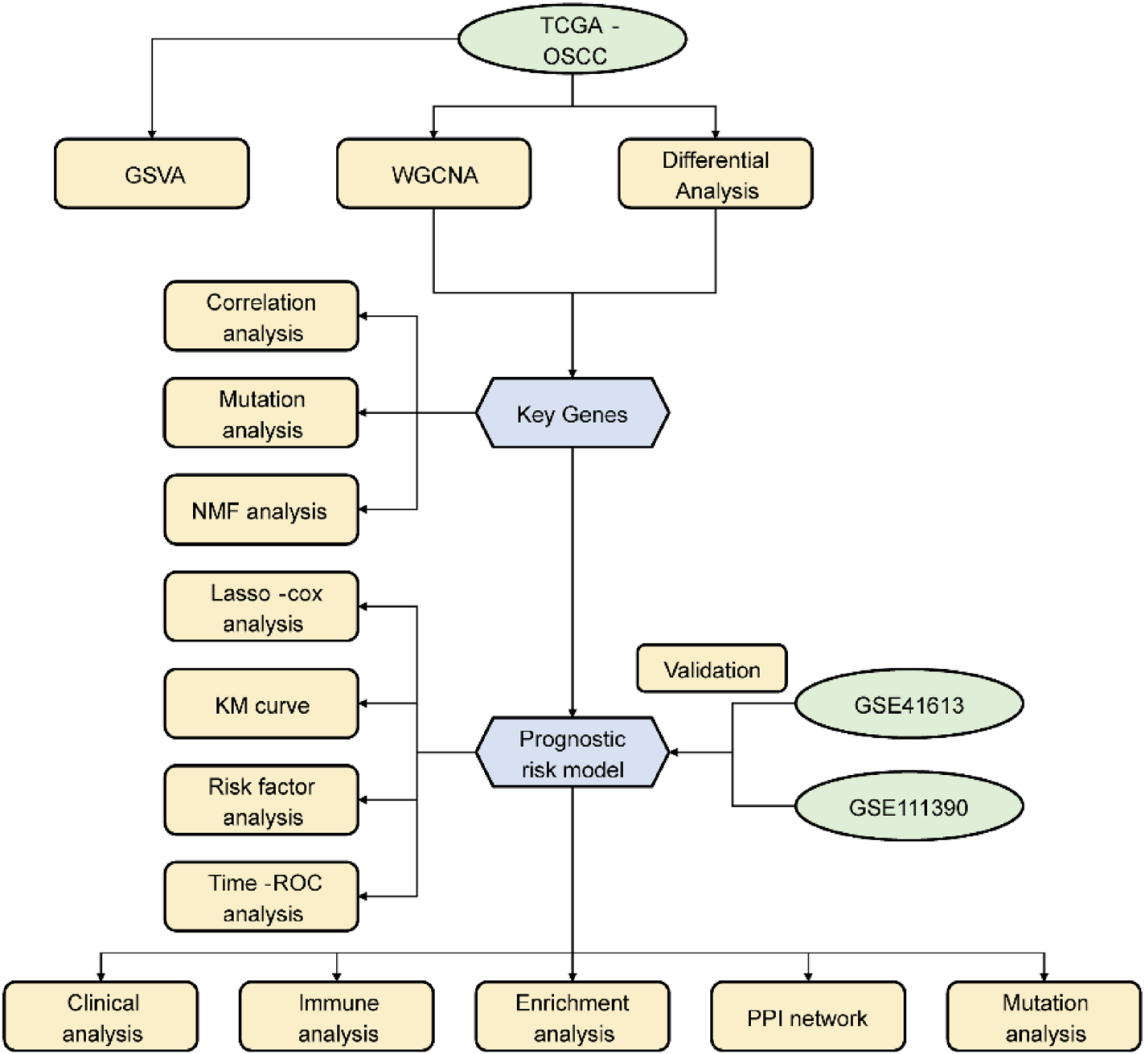
Outline of the current research.

**Figure 2:**
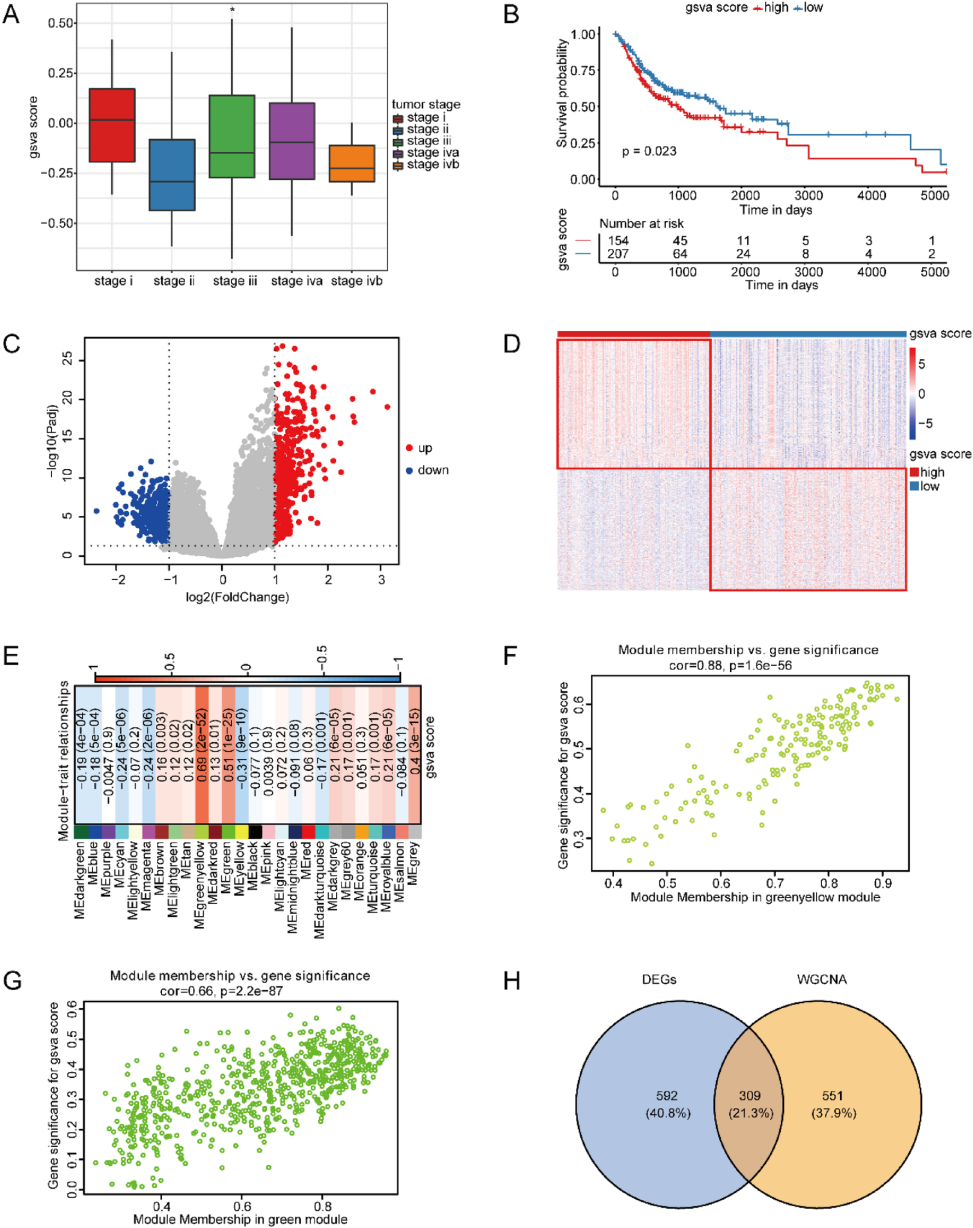
Identification of key genes in OSCC. (**A**) The relationship between GSVA proptosis score and tumor staging. *p < 0.05. (**B**) GSVA pyro ptosis score and KM survival curve. (**C**) DEGs volcano plot. The x-axis is log2 (Fold Change), the y-axis is − log10 (pad), and per dot denotes a gene. Blue dots denote down-regulated genes, red dots denote up-regulated genes, additionally, gray dots denote genes without significance. (**D**) Heat map of DEGs. The upper color bar represents two sets of samples. One set is blue, denoting the low GSVA pyroptosis scoring group. Besides, another one is red, denoting the high GSVA pyroptosis scoring group. (**E**) Heatmap showing the relationship between traits and modules. Every one of rows represents a gene module, and every column denotes a GSVA pyroptosis score of the sample predicted by the diagnostic model. Every cell contains the related relationship and *p*-value; red refers to the positive association, whereas blue refers to the negative association. (**F**) Module membership (MM) and Scatter plot of gene significance (GS) in the green yellow module. MM represents the correlation between module signature genes and the expression of genes. Genes in each module are highly correlated with the assigned module, indicating a high degree of connection within the module. GS denotes the absolute value of the association between genes and characteristics. Each dot in the graph represents a gene. The horizontal axis value represents the association between genes and modules, besides, the vertical axis value denotes the association between genes and traits. A highly significant association between GS and MM is shown, and genes highly correlated with traits are important elements in the modules that are correlated with this trait. (**G**) Scatter plot of GS and module membership (MM) in the green module. (**H**) The Venne diagram showing the cross-interaction of DEGs and module genes related to pyroptosis scores obtained from WGCNA.

To demonstrate the relationship between OSCC samples and pyroptosis at the transcriptomic level, we adopted the GSVA method, with the intention of calculating the pyroptosis score (gsva score) of the specimens (Fig. 1). We examined the relationship between pyroptosis score and tumor staging, and found that the pyroptosis score of stage II was the lowest and the most significant (Fig. 2A). Next, to exhibit the worse survival of the high scoring group, we detected the distinction between survivals of high and low scoring groups (Fig. 2B). Furthermore, the analysis on differentially express genes (DEGs) was implemented to indicate the biological distinctions between those groups in OSCC. After screening with statistical significance thresholds (Padj < 0.05, |logFC|> = 1), 901 DEGs in total were attained. In addition, 462 genes of them were up-regulated whereas 439 genes were down-regulated (Fig. 2C,D). WGCNA is capable of exploring the gene modules connected to the risk score of pyroptosis predicted by diagnostic models, and in the same module the genes may potentially to work jointly. Therefore, we performed WGCNA to identify the gene set related to pyroptosis score, and found that a high degree of correlation and significance was shown in greenyellow and green modules with the pyroptosis score (Fig. 2E–G). Among these modules, there were 171 genes in the greenyellow module as well as 689 genes in the green module. 309 intersection genes of the DEGs with the genes in greenyellow and green modules were considered as key genes associated with OSCC (Fig. 2H), including 304 up-regulated genes and 5 down-regulated genes.

**Figure 3:**
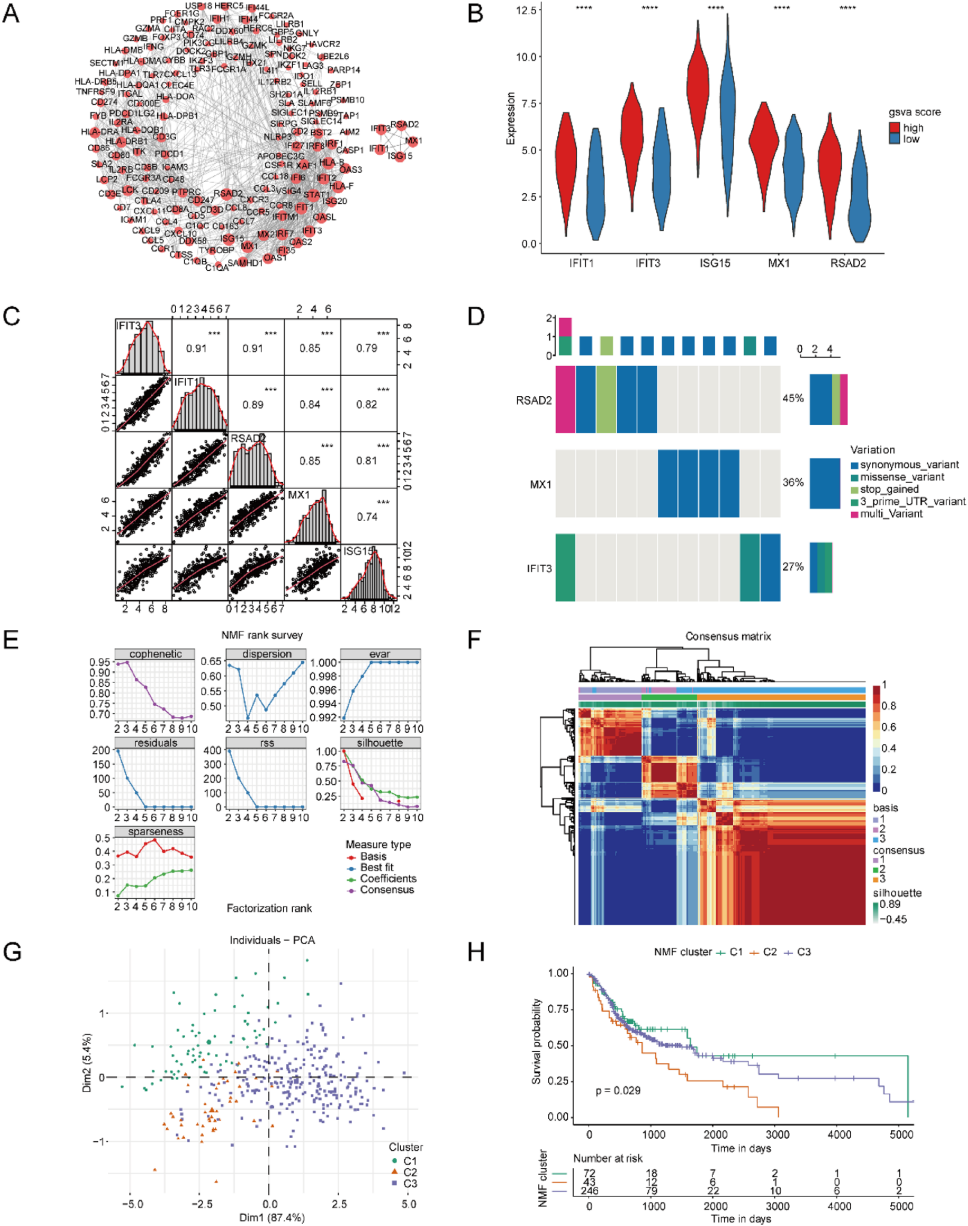
Characteristics of key genes in OSCC. (**A**) Left, PPI image of pyroptosis-related genes obtained from intersection, where the size of nodes is positively correlated with their degree; right, 5 hub gene subnetwork diagrams. (**B**) The differential expression of hub genes in high-risk and low-risk pyroptosis scoring groups. ****p < 0.0001. (**C**) Pearson correlation of hub genes. ***p < 0.001. (**D**) The mutation features of the hub genes. Upper bar chart, the type and frequency of mutations per specimen; right bar chart, type and frequency of mutations per gene. (**E**) Graph of the relationship between NMF clustering cophenetic correlation coefficient and cluster number. The horizontal axis denotes the cluster number between 2 and 10, besides, the vertical axis denotes the cophenetic coefficient. The optimal quantity of clusters is the quantity of clusters corresponding to a sharp decrease in the cophenetic coefficient. (**F**) NMF clustering. Blue to red represents the size of the connectivity values among samples. The larger the correlation value, the greater the probability of the samples clustering into one category. (**G**) PCA analysis of NMF clustering data. The x-axis and the y-axis denote two dimensionality reduction dimensions, additionally, each dot within the graph denotes a specimen. Green represents C1 class (Cluster 1) in the NMF clustering results, orange represents C2 class (Cluster 2), and purple represents C3 class (Cluster 3). (**H**) KM survival curve analysis of NMF clustering results.

A protein–protein interaction (PPI) network of 309 DEGs about pyroptosis was established by means of the STRING database which contained 262 nodes and 776 edges. To further analyze the characteristics of this network, hub nodes for the PPI network were mined, and the TOP5 genes of MCC score were identified as hub genes, namely IFIT1, IFIT3, ISG15, MX1 and RSAD2 (Fig. 3A), all of which were up-regulated genes (Fig. 3B). The correlation of hub genes was calculated, and it was found that the correlation score of all hub genes was above 0.7, which was prominent (*P* < 0.05; Fig. 3C). Specifically, the score between IFIT3 and IFIT1 was as high as 0.9. We also detected the hub gene mutation, and discovered that mutations only emerged in RSAD2, MX1, and IFIT3 genes, with each gene having synonymous mutations (Fig. 3D). Then, we conducted NMF unsupervised clustering based on hub genes. Furthermore, all specimens were split into three categories (C1, C2, and C3; Fig. 3E,F). PCA analysis of the NMF categories showed significant partitioning of the three categories of samples on two-dimensional coordinates, indicating the differences in the global transcriptome among the three categories (Fig. 3G). Survival analysis of Kaplan Meier (KM) exhibited the worst survival in C2, and the best survival in C1 (Fig. 3H).

### Prognostic model based upon key genes in OSCC

**Figure 4:**
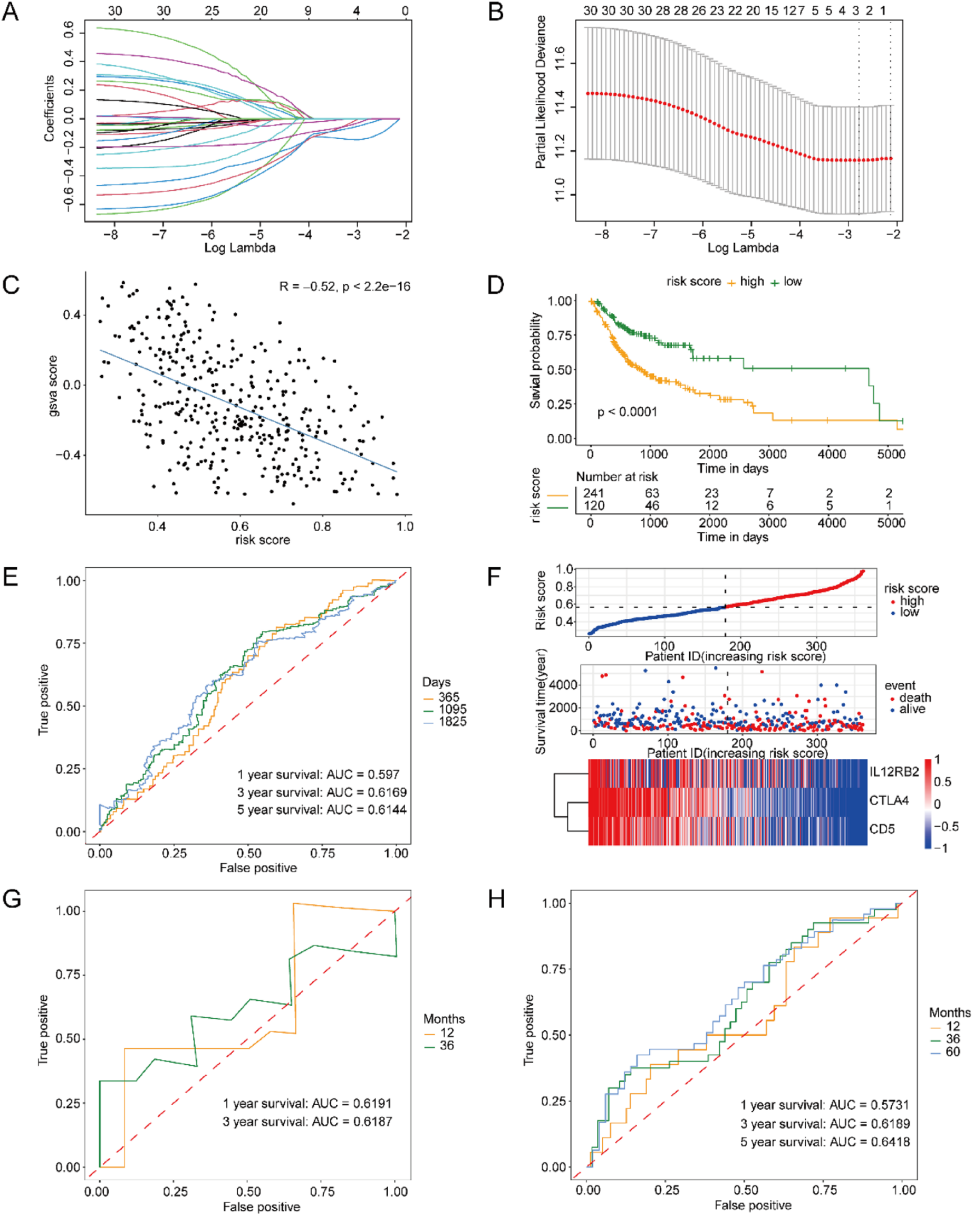
Construction and assessment of diagnostic models. (**A**) The LASSO regression curve showing the convergence screening process of LASSO regression for gene features. The x-axis refers to log lambda values, besides, the y-axis refers to regression coefficients. Lines of different colors represent different features. (**B**) The coefficient of LASSO-cox regression analysis. The horizontal axis refers to the tuning parameter (λ), the vertical axis refers to the binomial deviation, and the vertical black line refers to the optimal logλ value. (**C**) The association between GSVA pyroptosis score and risk score and. (**D**) The difference in survival curves between high-risk and low-risk groups. (**E**) ROC analysis and corresponding AUC for 1 year’s, 3 years’, and 5 years’ survival. (**F**) Risk factor analysis on prognostic model genes. (**G**,**H**) ROC analysis of the datasets GSE111390 (**G**) and GSE41613 (H) for verification. The x-axis refers to specificity, besides, the y-axis refers to sensitiveness.

With a view to constructing the research results into actual clinical applications, a prognostic model was established based upon key pyroptosis-related genes of OSCC, which is capable of forecasting the prognosis of the samples using the expression values of the key genes. Firstly, the univariate regression analysis was adopted, for the purpose of screening the genes linked with prognosis. Then, LASSO-cox regression was employed to screen for differentially expressed pyroptosis-related genes (DEPGs) connected to prognosis, retaining 3 genes (CTLA4, CD5, and IL12RB2) with a coefficient other than 0 (Fig. 4A,B). A multivariate prognostic model on the basis of these genes was set up (Fig. 3B). The association between risk score and pyroptosis score was figured out (Fig. 4C), with risk score = − 0.1365 * exp (CTLA4)—0.0322*exp (CD5)—0.0037*exp (IL12RB*2*). The result indicated a negative association between risk and pyroptosis scores, with a correlation score of − 0.52. With a view to verifying the evaluation efficiency of the diagnostic model, KM analysis was conducted based on the original dataset TCGA OSCC, and besides, the poor prognosis was in the high-risk group (Fig. 4D). Next, the predicted ROC was described, and the AUC was figured out. It was found that the predictive model had a great performance in both datasets, with the approximate 1-year, 3-years, and 5-years AUC (Fig. 4E). The risk factor regression analysis on the prognostic genes was performed. Additionally, it was found that the high expression of the three genes was linked with the low-risk group (Fig. 4F). ROC consequences of the validation datasets GSE111390 (Fig. 4G) and GSE41613 (Fig. 4H) displayed an AUC of approximately 0.6. Due to the limitation of data volume, we could not predict a 5-years survival rate based on dataset GSE111390.

### Gene enrichment analysis

**Figure 5:**
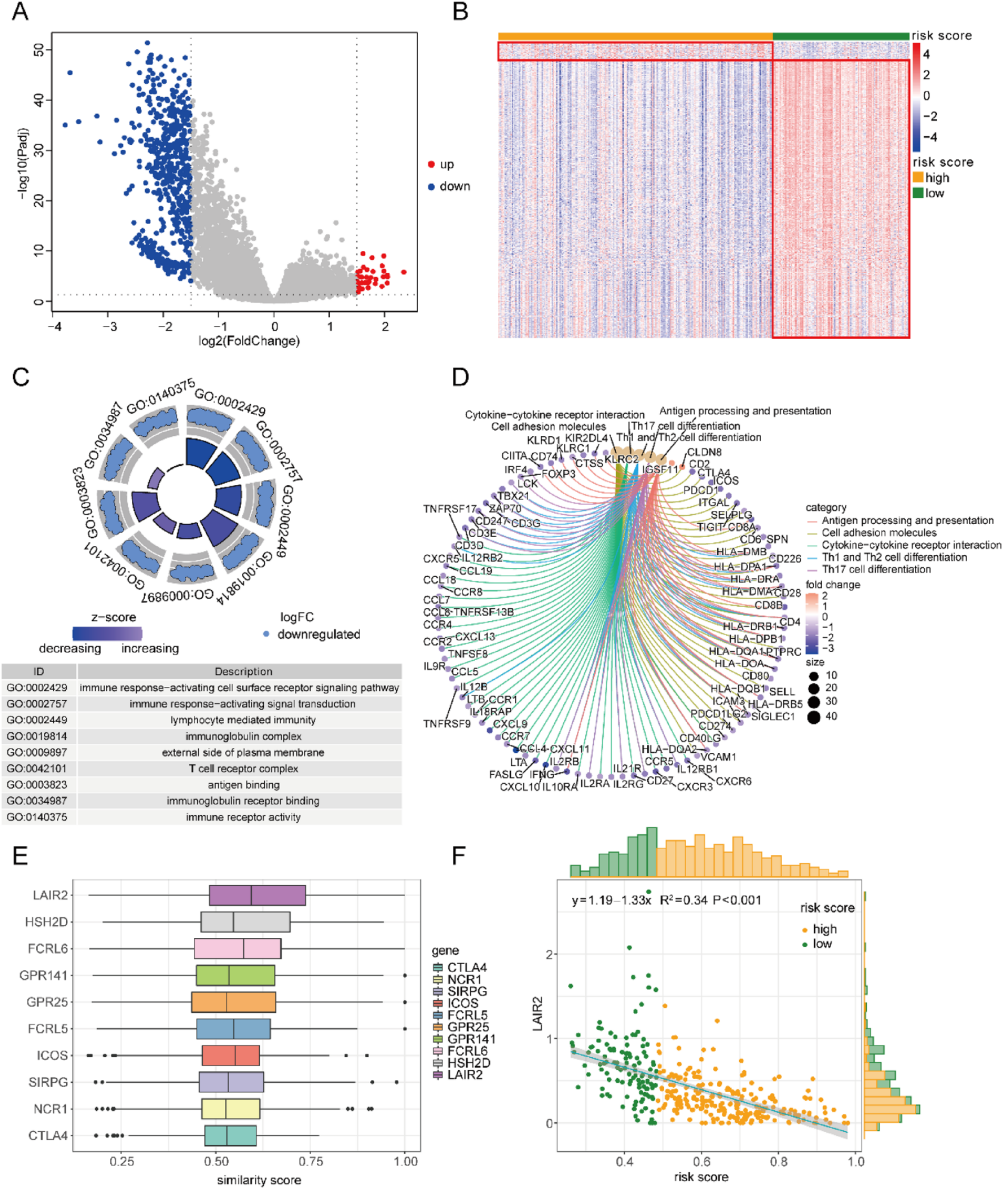
GO and KEGG enrichment analysis of DEGs. (**A**) DEGs volcano plot. The x-axis means log2 (Fold Change), the y-axis means − log10 (padj), and each dot refers to a gene. Blue dots refer to down-regulated genes, red dots refer to up-regulated genes, and gray dots refer to genes without significance. (**B**) DEGs heat map. The upper color bar represents two sets of samples, with green representing the low-risk scoring group and yellow representing the high-risk scoring group. (**C**) In the light of GO analysis, donut chart shows the enrichment analysis on TOP3 BP, CC, and MF terms. The outermost ring on the left refers to the GO term ID, the middle ring refers to the up-regulated genes, with each point representing a gene in GO term, and the innermost ring color refers to the z-score. If the gene is closer, the degree of down-regulation will be greater. The color bar length refers to the corrected *p*-value. (**D**) Donut chart showing the subordinate relationship between KEGG enriched pathways and genes. Genes are connected to their corresponding pathways by a string. The lower half circle represents the gene, besides the upper half represents the pathway. The gene color represents the logFC value. Different pathways are distinguished by different colors. (**E**) The TOP10 genes obtained through Friends analysis. The horizontal axis represents the similarity score with other genes based on semantic similarity. The score is positively correlated with the correlation with other genes, and genes with higher score are more important and can be considered as hub genes. (**F**) Scatter plot showing the similarity between hub genes and risk scores. Each dot represents a patient specimen, with yellow denoting the high-risk group and green denoting the low-risk group. The line represents the association fitting curve, the shaded area represents the confidence interval (CI), besides, the corresponding histogram is on the outer side of the graph.

To distinguish the expression differences between those two groups, the differential expression analysis was implemented, and 580 DEGs (|logFC|> 1.5, Padj < 0.05) was obtained, with 36 up-regulated and 543 down-regulated genes (Fig. 5A,B). With a view to deeply revealing the biological roles and courses influenced by DEPGs, GO enrichment analysis alongside KEGG enrichment analysis was visualized through different ways. It was shown in the GO enrichment results that the DEGs were largely associated with lymphocyte mediated immunity, immune response-activating cell surface receptor signaling pathway, and other immune-related pathways (Fig. 5C; Table S3). It was shown in the KEGG enrichment results that the DEGs were also associated with various courses, including antigen processing and presentation, Th1 and Th2 cell differentiation, and Th17 cell differentiation (Fig. 5D; Table S4). After identifying the hub genes of DEGs as *LAIR2* through Friends analysis (Fig. 5E), a passive relationship between *LAIR2* and the risk score were discovered (Fig. 5F).

**Figure 6:**
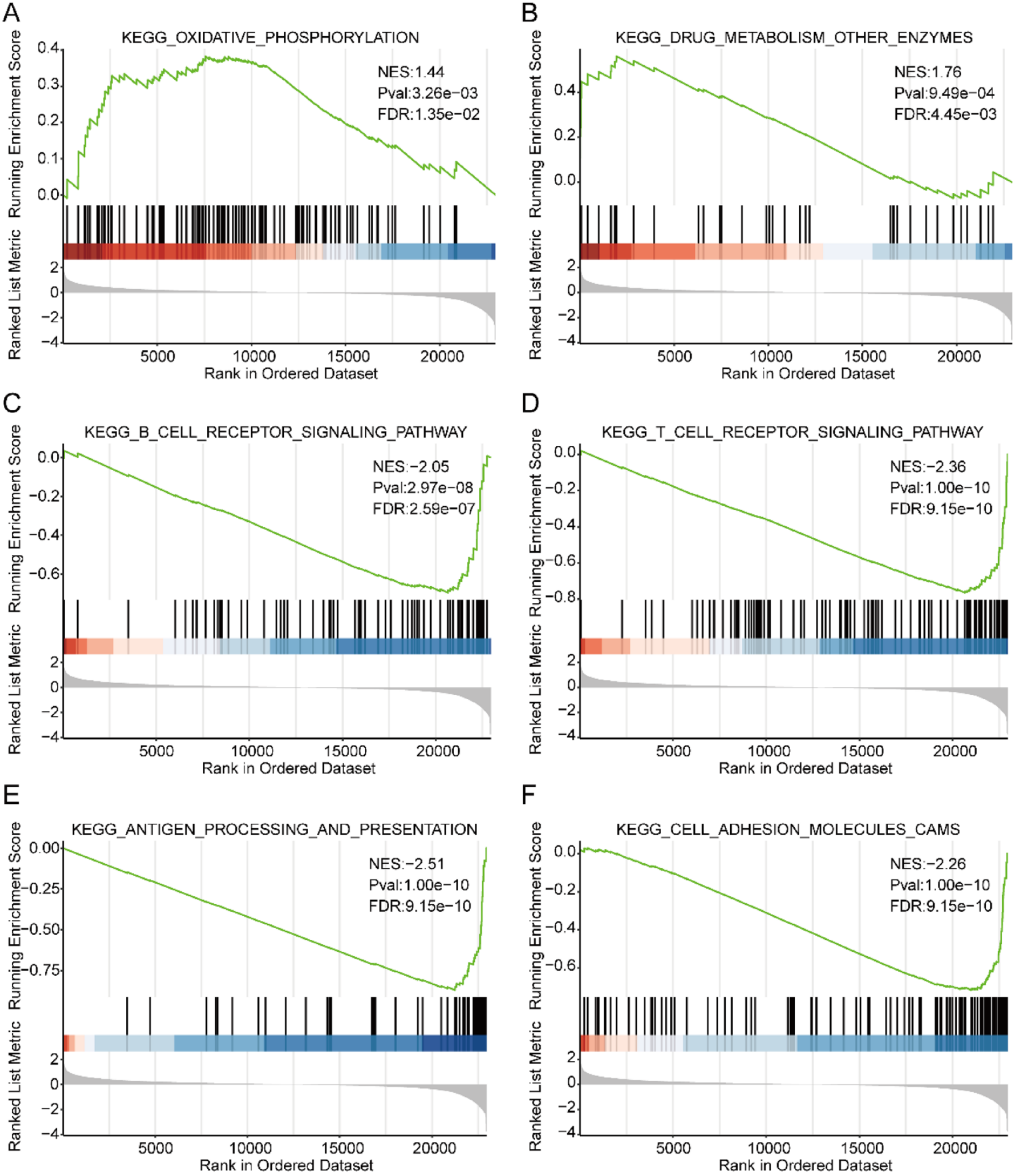
GSEA analysis. (**A**–**F**) Graphs showing the results of GSEA enrichment analysis of different terms: KEGG_OXIDATIVE_PHOSPHORYLATION (**A**), KEGG_DRUG_MATABOLISM_OTHER_ENZYMES (**B**), KEGG_B_CELL_RECEPTOR_SIGNALING_PATHWAY (**C**), KEGG_T_CELL_RECEPTOR_SIGNALING_PATHWAY (**D**), KEGG_ANTIGEN_PROCESSING_AND_PERSENTATION (**E**), KEGG_CELL_ADHESION_MOLECULES_CAMS (**F**). The x-axis refers to the rank of genes in the DEGs list, besides, the y-axis refers to the enrichment score.

With a view to deeply confirming the results of the enrichment analysis, GSEA method was adopted, with the aim of performing enrichment analysis on all DEGs based upon the KEGG background gene set. The results indicated that, in comparison with the low-risk group, the high-risk group was linked with metabolism-related functions, including the terms KEGG-OXIDATIVE_PHOSPHORYLATION and KEGG_ DRUG_ METABOLISM_ Other_ ENZYMES (Fig. 6A,B; Table S5). Prior researches have unraveled the specific correlation between oxidative stress damage and oral diseases^48^, and mitochondrial fusion could mediate oxidative phosphorylation, promoting the immortalization of cancer cells^49^. The immune function of the high-risk group was weakened by comparison with the low-risk group, regarding the terms such as KEGG_ B_ CELL_ RECEPTOR_ Signaling_ PATHWAY, KEGG_T_CELL_RECEPTOR_Signaling_PATHWAY, KEGG_ANTIGEN_ PROCESS_AND_PRESENTATION and KEGG_CELL_ADHESION_ MOLECULES_ CAMS (Fig. 6C–F). These results illustrated that the higher metabolic activity was found in the high-risk group, but the immune response was lower than that of the low-risk group.

### mRNA-miRNA interaction network

**Figure 7:**
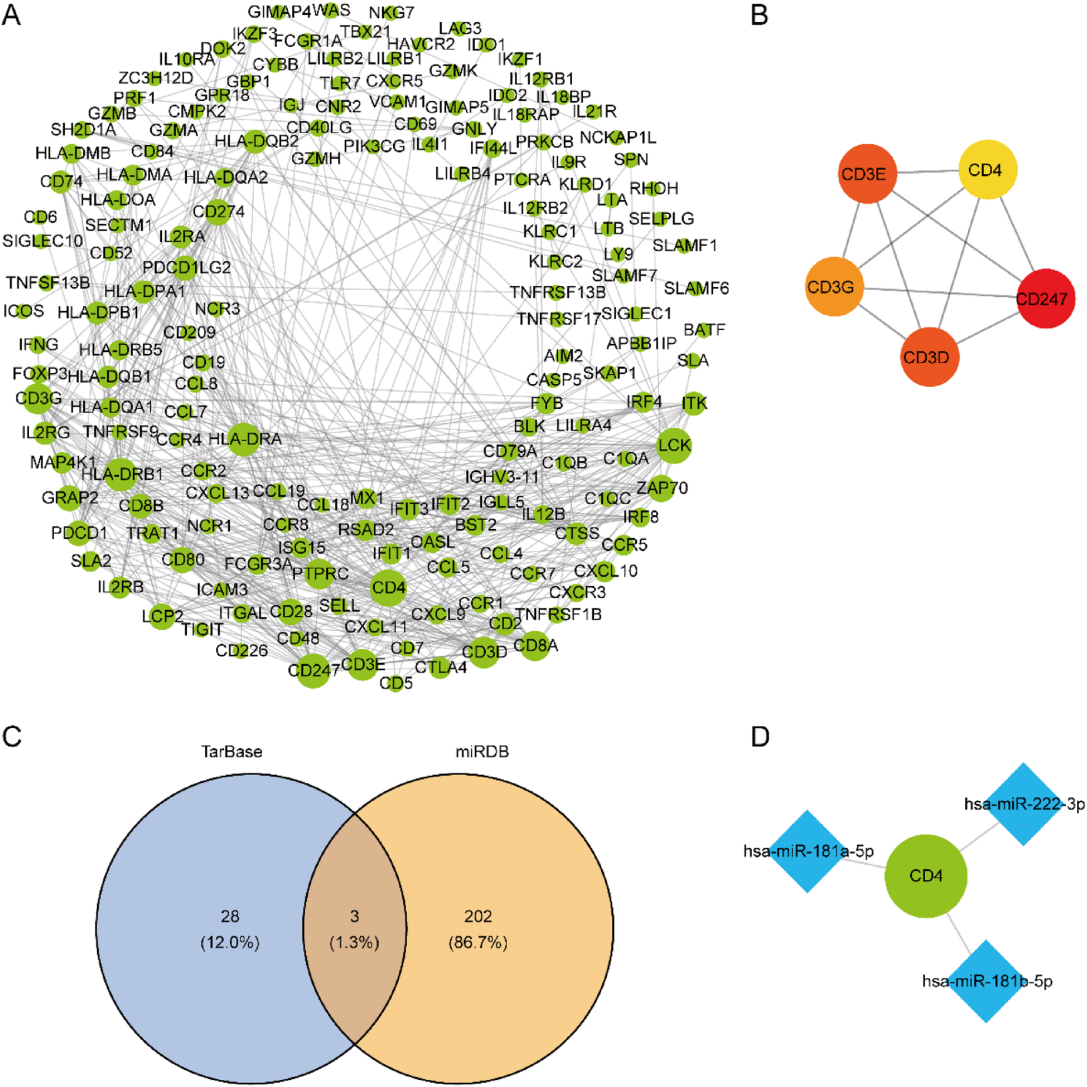
PPI network identifies key genes in OSCC. (**A**) DEGs PPI network in high-risk and low-risk groups, and the dot size represents the degree of nodes. (**B**) Subnetwork diagrams of 5 hub genes. The color between yellow and red refers to the MCC score from small to large. (**C**) Intersection of mRNA-miRNA pairs of hub genes in the TarBase and miRDB database. (**D**) mRNA-miRNA interaction network. Blue represents miRNA, and green represents hub genes.

The interactions between non-coding RNAs are mainly discovered through biological experiments, but such approaches are usually time-consuming and labor-intensive. These years, much progress has been made about the interaction prediction methods in various fields of computational biology^50–56^, which may provide valuable insights for the identification of genetic markers and non-coding RNAs though omics and multi-omics data related to OSCC. With a view to further investigating the correlation among DEPGs and analyzing the few key genes (hub genes) that play an important regulatory role, we constructed and visualized a PPI network and further analyzed the characteristics of the network. This network contained 362 genes that were highly correlated with both OSCC and pyroptosis, consisting of 563 edges (Fig. 7A). Next, we explored the hub genes in the network and extracted TOP5 nodes of MCC score as hub genes, including CD4, CD3E, CD3G, CD3D, and CD247 (Fig. 7B), which were considered as key genes related to OSCC and pyroptosis. In order to deepen our understanding of the OSCC transcriptome, we investigated the regulatory expression modes of the genes according to the mRNA-miRNA regulatory network. On the basis of five key genes related to OSCC and pyroptosis, three mRNA-miRNA pairs and three miRNAs, including miR-181a-5p, miR-181b-5p and miR-222-3p, which communicated with the key genes were identified through the Tarbase and miRDB database (Fig. 7C,D).

### The relationship between key genes in OSCC and immune infiltration

**Figure 8:**
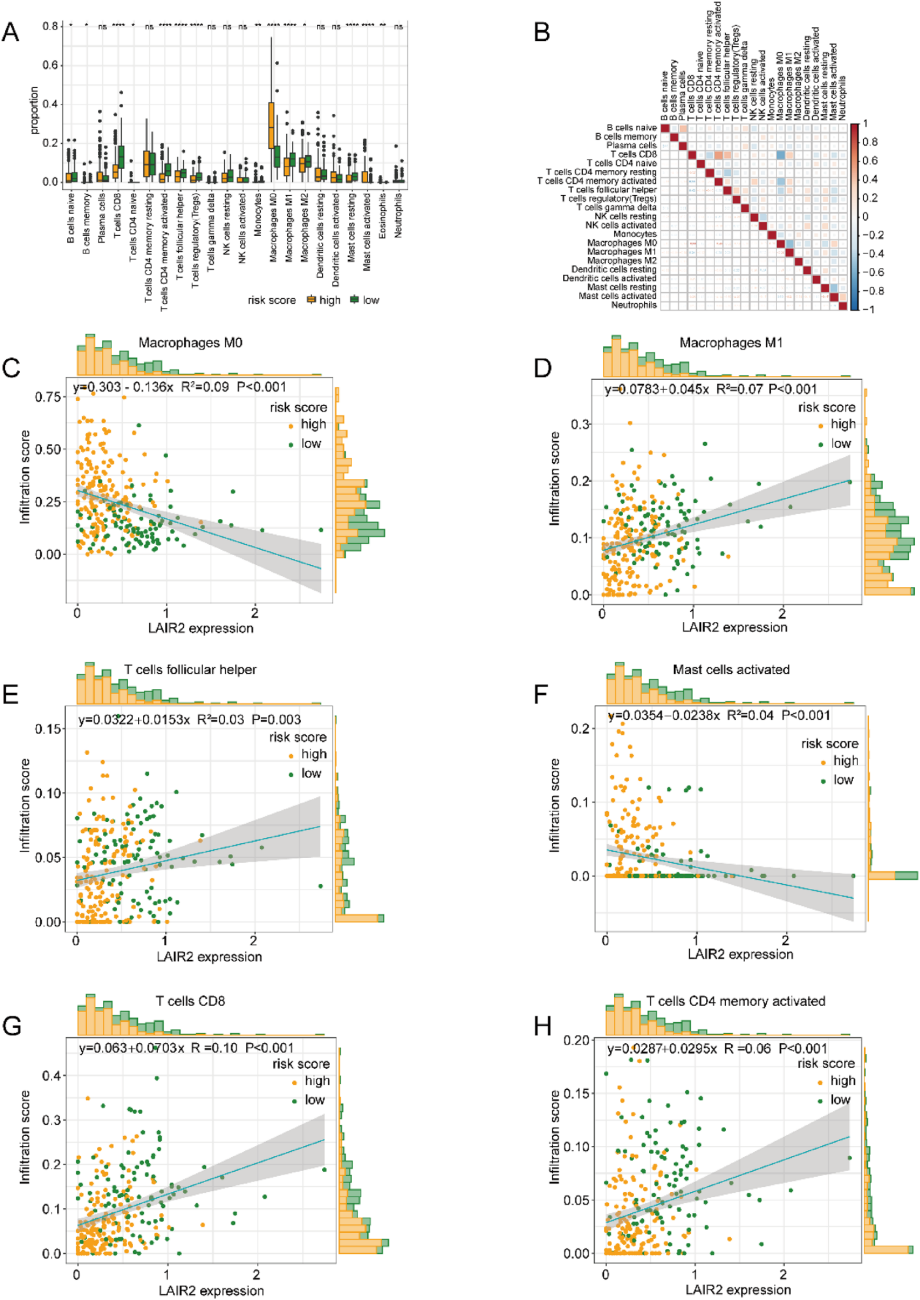
Immune infiltration analysis. (**A**) Distinctions of the levels of immune infiltration between two groups. The x-axis indicates immune cells, and the y-axis indicates the immune infiltration level. *p < 0.05, ** *p* < 0.01, *** *p* < 0.001, ****p < 0.0001, and ns and unsigned represent no significance. (**B**) Correlation analysis on immune cells. Red indicates positive association, while blue indicates negative association. (**C**–**H**) Scatter plots showing the association between the expression value of key gene *LAIR2* and macrophages M0 (**C**), macrophages M1 (**D**), T cells follicular helper (**E**), mast cells activated (**F**), T cells CD8 (**G**), and T cells memory activated (**H**). The x-axis indicates the mean *LAIR2* expression, besides, the y-axis indicates the level of immune cell infiltration. Each dot indicates a patient specimen, with yellow indicating the high-risk group and green indicating the low-risk group. The line represents the correlation fitting curve, the shaded area represents the CI, additionally, the corresponding histogram is on the outer side of the graph.

Next, the relationship between key genes related to OSCC and pyroptosis would be explored, as well as the level of immune cell infiltration. CIBERSORT was employed, with a view to calculating the immune cell infiltration scores of all samples with a gene set of 22 categories of immune cells. It was found that 14 out of 22 categories of immune cells showed significant variations in risk scores of pyroptosis between high and low scoring groups (Fig. 8A). Among them, T cells CD4 naive, monocytes, macrophages M0, mast cell activated, and eosinophils were more expressed in the pyroptosis high-risk group, whereas B cells naive, B cell memory, T cells CD8, T cells CD4 memory activated, T cells follicular helper, T cells regulatory (Tregs), macrophages M1, macrophages M2, and mast cell resting were more expressed in the group with low risk. By analyzing the correlation among various types of infiltrating cells, we observed a positive correlation among T cells CD8, T cells CD4 memory activated and T cells follicular helper (Fig. 8B).

In order to directly reveal the association between the expression of the hub gene *LAIR2* and the level of immune cell infiltration, we drew a scatter plot to show the significantly disparate immune cells and the *LAIR2* expression, and fitted the correlation curve (Fig. 8C–H). We observed that the *LAIR2* expression was passively associated with mast cells activated and macrophages M0, while it was proactively linked to macrophages M1, T cells follicular helper, T cells CD8, and T cells CD4 memory activated.

### Mutation signature analysis

**Figure 9:**
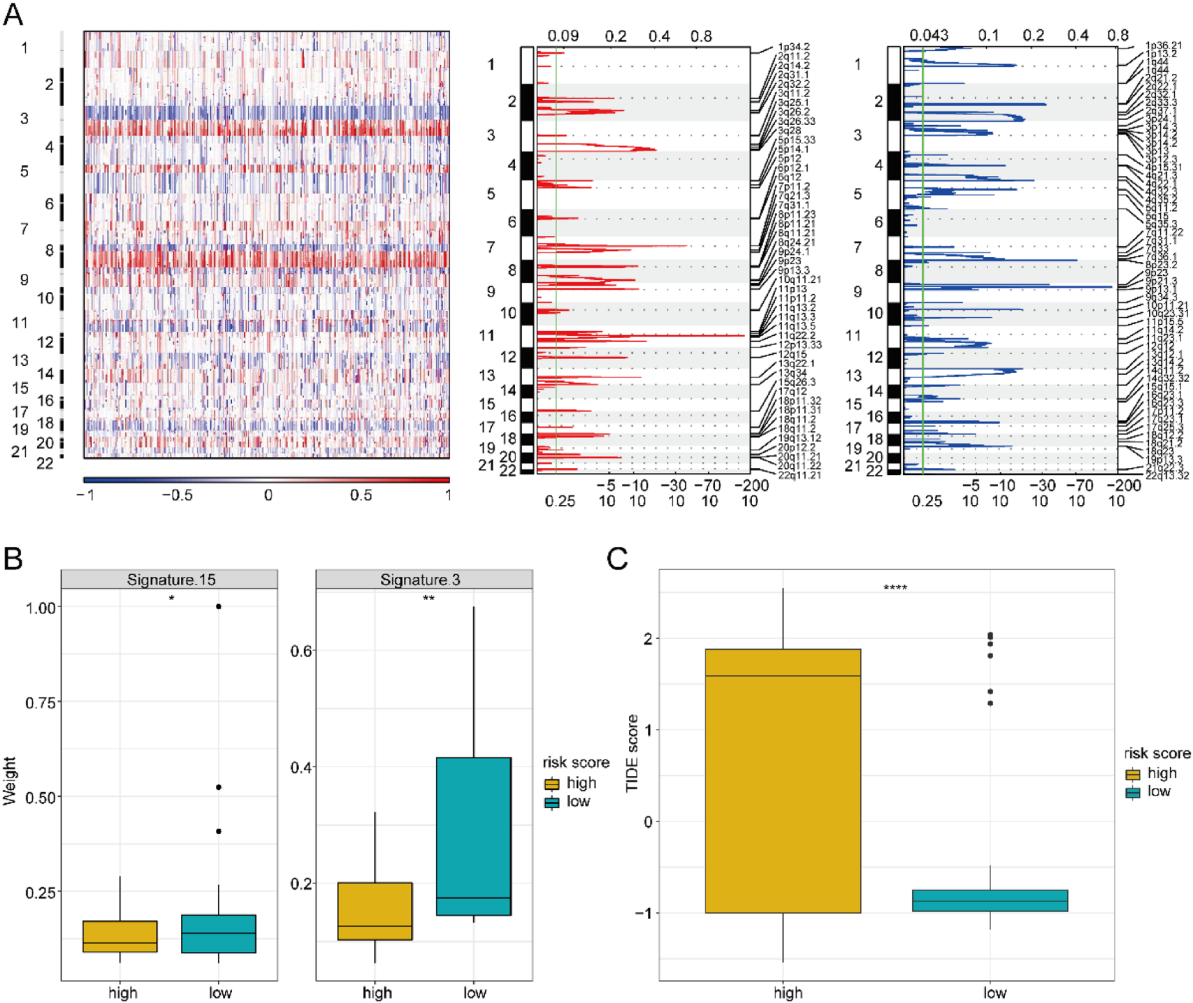
Mutation signature analysis. (**A**) GISTIC2 analysis identifying genes with prominent deletion or amplification. The error detection rate (Q-value) and GISTIC2.0 change score (x-axis) consist with the genome position (y-axis). The dashed line represents the centromere. The green one represents the 0.25 Q-value cutoff point to determine the significance. (**B**) The mutation signature of significant differences between two groups. *p < 0.05 and ** *p* < 0.01. (**C**) The difference of TIDE scores between two groups. ****p < 0.0001.

Tumor is mainly caused by the gradual acquisition of somatic genetic changes, such as point mutations and copy number changes, which affect the function of key genes regulating cell growth and survival. We adopted GISTIC 2.0, with the aim of identifying the genes exhibiting the features of prominent deletion or amplification (Fig. 9A). To deeply investigate the association between high-risk and low-risk groups as well as the treatment responses, signature nature 2013 were chosen as a known signature to infer the mutation differences between those two groups (Fig. 9B). It was discovered that signature.3 and signature.15 exceeded those in low-risk group. In addition, we examined the distinction in Tumor Immune Dysfunction and Exclusion (TIDE) scores between those groups, which serve as an alternative biomarker predicting the response to the immune checkpoint blockade. And we discovered the higher TIDE scores in high-risk group (Fig. 9C), suggesting the unsatisfactory response to immunotherapies of the high-risk group.

### Correlation between clinical factors and prognosis

**Figure 10:**
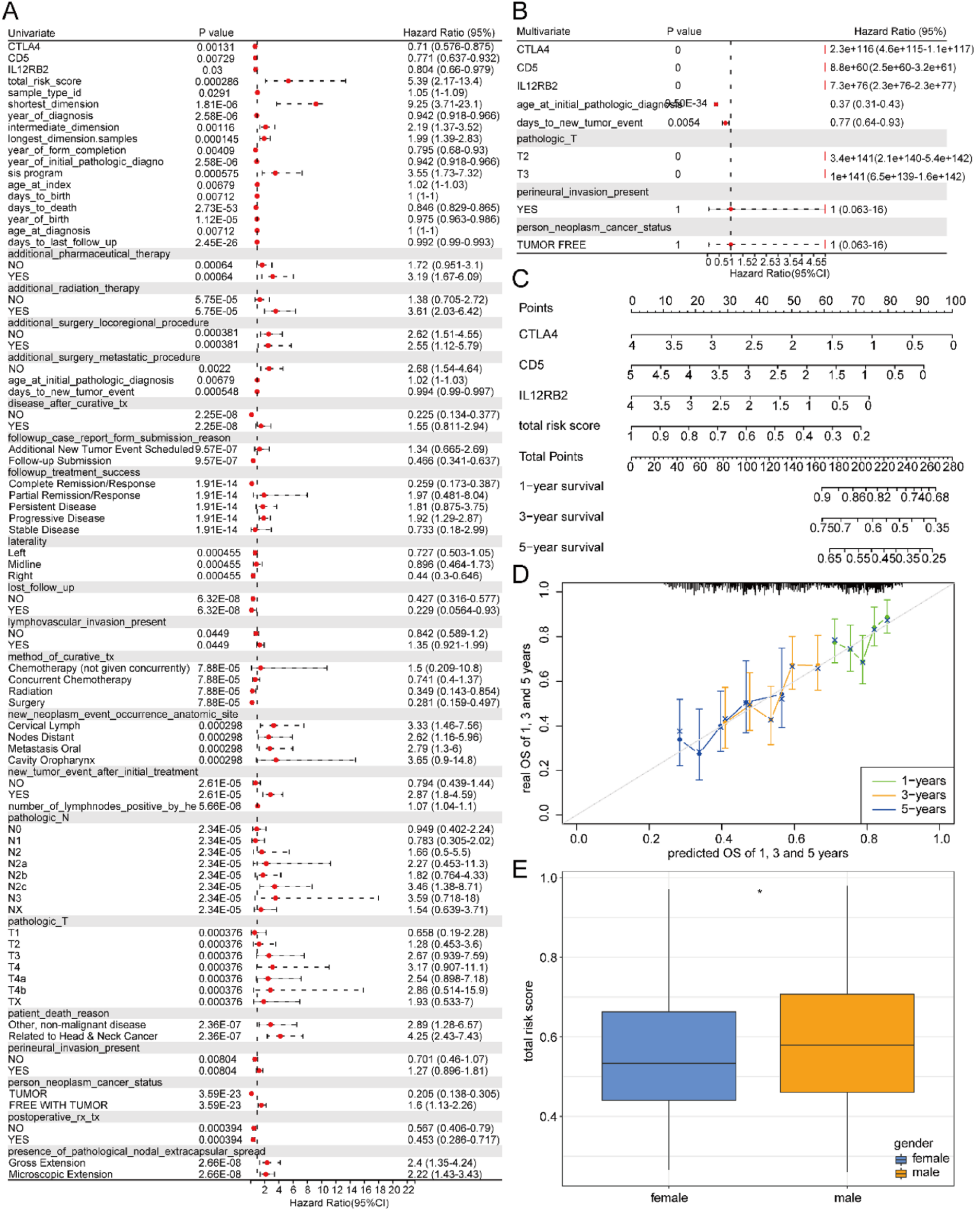
Correlation between clinical factors and prognosis. (**A**) Univariate regression analysis consequences. (**B**) Multivariate regression analysis consequences. (**C**) Nomogram analysis showing the relationship between prognostic genes and 1 year’s, 3 years’, and 5 years’ survival rates. (**D**) Calibration correction chart showing the correlation between predicted 1 year’s, 3 years’, and 5 years’ survival and actual 1 year’s, 3 years’, and 5 years’ survival. (**E**) The relationship between clinical factor gender and risk score. * *p* < 0.05.

Then, it was detected whether clinical characteristics were associated with prognosis. Univariate Cox regression analysis exhibited a significant correlation among prognostic model genes, staging, and overall survival (OS; Fig. 10A; Table S6). The univariate prognostic variables served as covariates to analyze the multivariate cox regression, indicating that the total three genes in the prognostic model were independent prognostic factors linked with OS (Fig. 10B; Table S6). With a view to assessing if our model was capable of precisely forecasting the prognosis of patients, the factors linked to OS were incorporated into the model. Besides, a column chart predicting the 1-year, 3-years, and 5-years patient OS rates was set up (Fig. 10C). Once again, the column chart model confirmed the dependability and clinically promising practicality of the risk model. Simultaneously, we conducted Calibration correction on the column chart and found a high correlation between the predicted results and the authentic survival ratio (Fig. 10D), suggesting accurate and reliable predicted results of this model. Finally, we performed a correlation analysis between gender and risk scores, and found risk scores were higher in males (Fig. 10E), which is consistent with a higher prevalence of OSCC in males compared to females according to a previous study^57^.

## Discussion

The morphological characteristics and molecular mechanisms of pyroptosis have been widely discussed. The function of pyroptosis in mediating host innate immune responses has also been revealed, but the role in anti-tumor immunity is yet elusive. In the current study, 309 genes were collectively identified as key pyroptosis-related genes in OSCC, and the PPI network of these genes was constructed. The univariate and LASSO-cox regression analysis was also employed to screen the DEPGs related to prognosis, identifying 3 important genes, namely CTLA4, CD5, and IL12RB2. Besides, the gene enrichment analysis on DEGs between high-risk and low-risk groups indicated that the metabolic activity was higher and the immune response was lower in the high-risk group.

CTLA4 is a family member of CD28 co-stimulatory molecules on T cells, which communicates with CD80 and CD86 on the surface of antigen-presenting or tumor cells, and thereby drives the activation or inhibition of T cell immune responses^58^. In the TCGA HNSC dataset, the higher expression of CD28 family proteins in the tumor microenvironment was associated with better overall survival, which contradicts the wide application of CTLA4 and PD-1 blockade for the immunotherapies of tumor^59^. Likewise, CD5 is a lymphoid-specific surface molecule expressed by all T cells and certain subtypes of B cells, triggering the intracellular signaling for cell activation or differentiation upon recognition of specific ligands^60,61^. It was reported that higher CD5 expression was connected to better prognosis in non-small cell lung cancer (NSCLC), probably benefiting from activating of tumor-specific T lymphocytes^62^. A recent study illustrated that the detection of CD5 + B cells in the tumor-draining lymph nodes represented lower staging in patients with HNSC^63^. However, the application of CD5 monoclonal antibody could promote the killing capacity of CD8 + T cells in mouse breast cancer, implying that CD5 blockade might be necessary to prevent the exhaustion of CD8 + T cells and therefore sustain the anti-tumor immunity^64^. In the current research, it was discovered that the higher CTLA4 and CD5 expression was associated with lower risk in OSCC prognosis. Considering the complex roles of both CD5 and CTLA4 in anti-tumor immunity, the practical application targeting CTLA4 or CD5 should be carefully investigated.

IL-12 is a kind of heterodimeric cytokine structurally associated with IL-23 and IL-27^65^. IL-12 initiates a series of intracellular responses predominantly mediated by STAT4^66^. IL12RB2 is a subunit of IL-12R, the receptor of IL-12. It was previously reported that *Il12rb2* gene could act as an anti-oncogene in human chronic B-cell cancers^67^, implying the potential role of IL12RB2 and IL-12/IL-12R signaling in anti-tumor immunity. *Il12rb2* knock-out mice was observed to be more susceptible to lung adenocarcinoma (LAC)^68^, and two allele variants of IL12RB2 increased the risk of LAC^69^. Another study identified four genes, including IL12RB2, ADAM15, CDC7, and TNFRSF8, as potential biomarkers to the diagnose and treat OSCC^70^, consistent with our identification of IL12RB2 in the current study. Therefore, a more comprehensive understanding of the specific functions of IL12RB2 as well as IL-12/IL-12R signaling in OSCC is needed in the following researches.

In the past decades, the relationship between pyroptosis and the development of cancer has attracted much attention. However, it is currently unclear how genes related to pyroptosis interact and whether they are associated with the survival of cancer patients. In our current study, by performing comprehensive bioinformatic analyses, we revealed the correlation between pyroptosis and the prognosis of OSCC for the very first time. We unraveled that expression of pyroptosis-related genes was linked to the OSCC survival rate, and established one prognostic model based upon three differentially expressed pyroptosis-related genes, namely CTLA4, CD5 and IL12RB2, which might serve as promising biomarkers and treatment targets for OSCC. Also, to some extent, our analysis of somatic mutations in OSCC is a supplement and innovation to previous studies, which may have guiding significance for clinical treatment and prognosis assessment.

There are still many challenges by targeting pyroptosis to clinically intervene tumors, because additional experiments, database of more samples, longer follow-ups are essential to further prove the conclusions in this study. The inconsistent rankings of pyroptosis-related genes in OSCC might be caused by the different database and the sample size, as well as the different feature weight calculation methods. Therefore, it must be admitted that the key genes identified in our current study need further validation. More in vitro and vivo trials need to be implemented at cellular and tissue levels so as to improve and develop therapeutic strategies. Besides, additional analysis approaches can be adopted to further verify our current findings or provide novel insights, such as ordinary differential equation (ODE) based modeling, which is a well-established approach to quantitatively study the cellular regulatory mechanism. A previous study has used ODE modeling to uncover the switching mechanisms among pyroptosis and apoptosis^71^, implying the potentiality of this approach in understanding the regulatory mechanisms and finding potential therapeutic targets in OSCC. Furthermore, considering that the current study was conducted based on publicly databases, further investigation about the applicability of our prognostic model for clinical trials is definitely needed.

### Supplementary Information

Supplementary Table S1.

Supplementary Table S2.

Supplementary Table S3.

Supplementary Table S4.

Supplementary Table S5.

Supplementary Table S6.

## Data Availability

All the resources are free to obtain online. The raw data is in https://pan.baidu.com/s/1IjNnzvv1iLs9Ah1D4IBOnA; cw6t.

## Abbreviations

AUC: Area under the curve
BH: Benjamin Hochberg
BP: Biological process
CC: Cellular component
DEG: Differentially expressed gene
DEPG: Differentially expressed pyroptosis-related gene
FDR: False discovery rate
GDC: Genomic data commons
GO: Gene otology
GSEA: Gene set enrichment analysis
GSVA: Gene set variation analysis
HNSC: Head and neck squamous cell carcinoma
HPV: Human papillomavirus
KEGG: Kyoto encyclopedia of genes and genomes
KM: Kaplan Meier
LAC: Lung adenocarcinoma
LAIR2: Leukocyte-associated Ig-like receptor-2
LASSO: Least absolute shrinkage and selection operator
LSCC: Laryngeal squamous cell carcinoma
MCC: McCreight
MF: Molecular function
NMF: Non-negative matrix factorization
NSCLC: Non-small cell lung cancer
OS: Overall survival
OSCC: Oral squamous cell carcinoma
PCA: Principal component analysis
PPI: Protein–protein interaction
PSCC: Primary squamous cell carcinoma
ROC: Receiver-operating curve
STRING: Search tool for the retrieval of interaction gene/proteins
TCGA: The Cancer Genome Atlas
TIDE: Tumor immune dysfunction and exclusion
TOM: Topological overlap matrix
WGCNA: Weighted correlation network analysis
