# Supplementary material for: Prognostic model revealing pyroptosis-related signatures in oral squamous cell carcinoma based on bioinformatics analysis
Source: Sci Rep. 2024 Mar 14;14:6149. doi: 10.1038/s41598-024-56694-y (PMC10937718; doi:10.1038/s41598-024-56694-y)
Supplement: Supplementary file 1 — Supplementary Table S1. [file 41598_2024_56694_MOESM1_ESM.docx]

**Table S1. Clinical Baseline Data**

|  | **TCGA-OSCC** | **GSE41613** | **GSE111390** |
| --- | --- | --- | --- |
| Organism | Homo sapiens | Homo sapiens | Homo sapiens |
| Experiment type | RNAseq | Expression profiling by array | Expression profiling by array |
| Data type | mRNA expression  copy number data  somatic mutation  phenotype data  clinical data | mRNA expression  clinical data | mRNA expression  clinical data |
| Sample Number | 362 (Tumor: 330, Normal: 32) | 97(Tumor: 97, Normal: 0) | 14（Tumor:14, Normal: 0） |
| Platforms | Illumina | GPL570 | GPL6480 |
| **Status** | | |  |
| Alive | 177 | 46 | 8 |
| Dead | 184 | 51 | 6 |
