# Supplementary material for: Prognostic model revealing pyroptosis-related signatures in oral squamous cell carcinoma based on bioinformatics analysis
Source: Sci Rep. 2024 Mar 14;14:6149. doi: 10.1038/s41598-024-56694-y (PMC10937718; doi:10.1038/s41598-024-56694-y)
Supplement: Supplementary file 2 — Supplementary Table S2. [file 41598_2024_56694_MOESM2_ESM.docx]

**Table S2. Pyroptosis-related genes**

| Gene | Gene | Gene | Gene | Gene | Gene |
| --- | --- | --- | --- | --- | --- |
| *AIM2* | *CHMP2B* | *GSDMB* | *CYCS* | *NLRP2* | *TIRAP* |
| *CASP1* | *BAX* | *GSDMC* | *CHMP6* | *NLRP3* | *TNF* |
| *CASP3* | *CHMP4B* | *GSDMD* | *HMGB1* | *NLRP6* | *GSMDC* |
| *CASP4* | *IL1A* | *GSDME* | *CHMP4A* | *NLRP7* | *GZMA* |
| *CASP5* | *CHMP3* | *IL18* | *PJVK* | *GPX4* | *CHMP4C* |
| *CASP6* | *IRF1* | *IL1B* | *PLCG1* | *GSDMA* | *IRF2* |
| *CASP8* | *CHMP2A* | *IL6* | *PRKACA* | *NOD1* | *GZMB* |
| *CASP9* | *TP53* | *NLRC4* | *PYCARD* | *NOD2* | *BAK1* |
| *ELANE* | *CHMP7* | *NLRP1* | *SCAF11* | *TP63* |  |
