# Supplementary material for: Prognostic model revealing pyroptosis-related signatures in oral squamous cell carcinoma based on bioinformatics analysis
Source: Sci Rep. 2024 Mar 14;14:6149. doi: 10.1038/s41598-024-56694-y (PMC10937718; doi:10.1038/s41598-024-56694-y)
Supplement: Supplementary file 3 — Supplementary Table S3. [file 41598_2024_56694_MOESM3_ESM.docx]

**Table S3. GO enrichment analysis**

| **ONTOLOGY** | **ID** | **Description** | **P adjust** |
| --- | --- | --- | --- |
| BP | GO:0002429 | immune response-activating cell surface receptor signaling pathway | 7.02E-113 |
| BP | GO:0002757 | immune response-activating signal transduction | 7.02E-113 |
| BP | GO:0002449 | lymphocyte mediated immunity | 6.37E-109 |
| BP | GO:0002460 | adaptive immune response based on somatic recombination of immune receptors built from immunoglobulin superfamily domains | 1.49E-103 |
| BP | GO:0002455 | humoral immune response mediated by circulating immunoglobulin | 1.99E-91 |
| BP | GO:0019724 | B cell mediated immunity | 2.70E-90 |
| BP | GO:0006958 | complement activation, classical pathway | 1.04E-89 |
| BP | GO:0016064 | immunoglobulin mediated immune response | 1.42E-89 |
| BP | GO:0006956 | complement activation | 6.96E-82 |
| BP | GO:0050851 | antigen receptor-mediated signaling pathway | 1.43E-79 |
| BP | GO:0051251 | positive regulation of lymphocyte activation | 3.97E-76 |
| BP | GO:0002696 | positive regulation of leukocyte activation | 4.40E-74 |
| BP | GO:0050867 | positive regulation of cell activation | 5.27E-74 |
| BP | GO:0006959 | humoral immune response | 7.78E-72 |
| BP | GO:0002697 | regulation of immune effector process | 2.54E-69 |
| BP | GO:0006909 | phagocytosis | 4.11E-68 |
| BP | GO:0050853 | B cell receptor signaling pathway | 6.00E-62 |
| BP | GO:0002440 | production of molecular mediator of immune response | 1.36E-60 |
| BP | GO:0050864 | regulation of B cell activation | 1.95E-59 |
| BP | GO:0002377 | immunoglobulin production | 1.51E-58 |
| BP | GO:0042113 | B cell activation | 1.28E-55 |
| BP | GO:0002920 | regulation of humoral immune response | 6.79E-55 |
| BP | GO:0030449 | regulation of complement activation | 1.79E-54 |
| BP | GO:0002431 | Fc receptor mediated stimulatory signaling pathway | 2.28E-53 |
| BP | GO:0050871 | positive regulation of B cell activation | 2.49E-52 |
| BP | GO:0002433 | immune response-regulating cell surface receptor signaling pathway involved in phagocytosis | 8.81E-52 |
| BP | GO:0038096 | Fc-gamma receptor signaling pathway involved in phagocytosis | 8.81E-52 |
| BP | GO:0038094 | Fc-gamma receptor signaling pathway | 1.40E-51 |
| BP | GO:0006910 | phagocytosis, recognition | 2.52E-51 |
| BP | GO:0006911 | phagocytosis, engulfment | 2.74E-50 |
| BP | GO:0099024 | plasma membrane invagination | 2.58E-48 |
| BP | GO:0042110 | T cell activation | 3.22E-48 |
| BP | GO:0010324 | membrane invagination | 1.01E-46 |
| BP | GO:0038093 | Fc receptor signaling pathway | 1.02E-43 |
| BP | GO:0008037 | cell recognition | 3.27E-42 |
| BP | GO:0038095 | Fc-epsilon receptor signaling pathway | 2.21E-38 |
| BP | GO:0050863 | regulation of T cell activation | 5.45E-38 |
| BP | GO:0042742 | defense response to bacterium | 1.43E-37 |
| BP | GO:0007159 | leukocyte cell-cell adhesion | 7.63E-34 |
| BP | GO:1903037 | regulation of leukocyte cell-cell adhesion | 1.05E-32 |
| BP | GO:1903131 | mononuclear cell differentiation | 1.21E-31 |
| BP | GO:0050870 | positive regulation of T cell activation | 8.06E-31 |
| BP | GO:1903039 | positive regulation of leukocyte cell-cell adhesion | 8.44E-31 |
| BP | GO:0030098 | lymphocyte differentiation | 1.18E-30 |
| BP | GO:0046651 | lymphocyte proliferation | 1.17E-29 |
| BP | GO:0032943 | mononuclear cell proliferation | 2.01E-29 |
| BP | GO:0070661 | leukocyte proliferation | 2.80E-29 |
| BP | GO:0070663 | regulation of leukocyte proliferation | 3.88E-29 |
| BP | GO:0006898 | receptor-mediated endocytosis | 4.55E-29 |
| BP | GO:0050670 | regulation of lymphocyte proliferation | 6.47E-29 |
| BP | GO:0032944 | regulation of mononuclear cell proliferation | 9.85E-29 |
| BP | GO:0030217 | T cell differentiation | 1.00E-28 |
| BP | GO:0022407 | regulation of cell-cell adhesion | 2.37E-28 |
| BP | GO:0022409 | positive regulation of cell-cell adhesion | 3.11E-28 |
| BP | GO:0042129 | regulation of T cell proliferation | 9.42E-27 |
| BP | GO:0042098 | T cell proliferation | 3.80E-26 |
| BP | GO:0042102 | positive regulation of T cell proliferation | 1.03E-23 |
| BP | GO:0050852 | T cell receptor signaling pathway | 3.02E-23 |
| BP | GO:0046631 | alpha-beta T cell activation | 3.63E-22 |
| BP | GO:0045785 | positive regulation of cell adhesion | 1.46E-21 |
| BP | GO:0050671 | positive regulation of lymphocyte proliferation | 1.78E-21 |
| BP | GO:0032946 | positive regulation of mononuclear cell proliferation | 2.26E-21 |
| BP | GO:0070665 | positive regulation of leukocyte proliferation | 3.31E-21 |
| BP | GO:0045619 | regulation of lymphocyte differentiation | 1.01E-18 |
| BP | GO:0001819 | positive regulation of cytokine production | 1.05E-18 |
| BP | GO:0002706 | regulation of lymphocyte mediated immunity | 2.88E-18 |
| BP | GO:0045058 | T cell selection | 4.28E-18 |
| BP | GO:0002703 | regulation of leukocyte mediated immunity | 4.75E-18 |
| BP | GO:0002683 | negative regulation of immune system process | 5.26E-18 |
| BP | GO:0032609 | interferon-gamma production | 2.23E-17 |
| BP | GO:0045580 | regulation of T cell differentiation | 3.06E-17 |
| BP | GO:0002695 | negative regulation of leukocyte activation | 3.06E-17 |
| BP | GO:0034341 | response to interferon-gamma | 4.43E-17 |
| BP | GO:0032649 | regulation of interferon-gamma production | 7.71E-17 |
| BP | GO:0043368 | positive T cell selection | 1.58E-16 |
| BP | GO:1902105 | regulation of leukocyte differentiation | 2.38E-16 |
| BP | GO:0050866 | negative regulation of cell activation | 5.63E-16 |
| BP | GO:0046632 | alpha-beta T cell differentiation | 9.21E-16 |
| BP | GO:0071346 | cellular response to interferon-gamma | 9.21E-16 |
| BP | GO:0031295 | T cell costimulation | 4.33E-15 |
| BP | GO:0002228 | natural killer cell mediated immunity | 7.79E-15 |
| BP | GO:0002699 | positive regulation of immune effector process | 8.68E-15 |
| BP | GO:0031294 | lymphocyte costimulation | 8.73E-15 |
| BP | GO:0046634 | regulation of alpha-beta T cell activation | 3.01E-14 |
| BP | GO:0001909 | leukocyte mediated cytotoxicity | 3.81E-14 |
| BP | GO:0002285 | lymphocyte activation involved in immune response | 4.58E-14 |
| BP | GO:0002715 | regulation of natural killer cell mediated immunity | 5.06E-14 |
| BP | GO:0051250 | negative regulation of lymphocyte activation | 8.04E-14 |
| BP | GO:0002819 | regulation of adaptive immune response | 1.51E-13 |
| BP | GO:0002286 | T cell activation involved in immune response | 2.52E-13 |
| BP | GO:0031349 | positive regulation of defense response | 2.52E-13 |
| BP | GO:0042267 | natural killer cell mediated cytotoxicity | 7.08E-13 |
| BP | GO:0002822 | regulation of adaptive immune response based on somatic recombination of immune receptors built from immunoglobulin superfamily domains | 8.87E-13 |
| BP | GO:1903706 | regulation of hemopoiesis | 1.19E-12 |
| BP | GO:0002708 | positive regulation of lymphocyte mediated immunity | 1.35E-12 |
| BP | GO:0031341 | regulation of cell killing | 1.74E-12 |
| BP | GO:0002831 | regulation of response to biotic stimulus | 1.74E-12 |
| BP | GO:0070098 | chemokine-mediated signaling pathway | 2.07E-12 |
| BP | GO:0001910 | regulation of leukocyte mediated cytotoxicity | 2.87E-12 |
| BP | GO:0002705 | positive regulation of leukocyte mediated immunity | 3.35E-12 |
| BP | GO:0001906 | cell killing | 3.79E-12 |
| BP | GO:0042269 | regulation of natural killer cell mediated cytotoxicity | 3.93E-12 |
| BP | GO:0043367 | CD4-positive, alpha-beta T cell differentiation | 4.51E-12 |
| BP | GO:0002833 | positive regulation of response to biotic stimulus | 5.29E-12 |
| BP | GO:0050868 | negative regulation of T cell activation | 9.92E-12 |
| BP | GO:0045088 | regulation of innate immune response | 1.01E-11 |
| BP | GO:0032729 | positive regulation of interferon-gamma production | 1.21E-11 |
| BP | GO:1990868 | response to chemokine | 1.33E-11 |
| BP | GO:1990869 | cellular response to chemokine | 1.33E-11 |
| BP | GO:0046635 | positive regulation of alpha-beta T cell activation | 1.55E-11 |
| BP | GO:0045089 | positive regulation of innate immune response | 2.05E-11 |
| BP | GO:0045582 | positive regulation of T cell differentiation | 2.05E-11 |
| BP | GO:0070664 | negative regulation of leukocyte proliferation | 2.05E-11 |
| BP | GO:0035710 | CD4-positive, alpha-beta T cell activation | 2.33E-11 |
| BP | GO:0060333 | interferon-gamma-mediated signaling pathway | 3.81E-11 |
| BP | GO:0050672 | negative regulation of lymphocyte proliferation | 4.75E-11 |
| BP | GO:0002456 | T cell mediated immunity | 4.92E-11 |
| BP | GO:0032945 | negative regulation of mononuclear cell proliferation | 5.84E-11 |
| BP | GO:0031343 | positive regulation of cell killing | 8.75E-11 |
| BP | GO:0002717 | positive regulation of natural killer cell mediated immunity | 1.64E-10 |
| BP | GO:0050854 | regulation of antigen receptor-mediated signaling pathway | 1.87E-10 |
| BP | GO:1903038 | negative regulation of leukocyte cell-cell adhesion | 1.91E-10 |
| BP | GO:0045621 | positive regulation of lymphocyte differentiation | 2.52E-10 |
| BP | GO:0002709 | regulation of T cell mediated immunity | 2.57E-10 |
| BP | GO:0009615 | response to virus | 3.76E-10 |
| BP | GO:0071674 | mononuclear cell migration | 5.44E-10 |
| BP | GO:0002292 | T cell differentiation involved in immune response | 9.30E-10 |
| BP | GO:0002507 | tolerance induction | 9.74E-10 |
| BP | GO:0045066 | regulatory T cell differentiation | 1.20E-09 |
| BP | GO:0006968 | cellular defense response | 1.34E-09 |
| BP | GO:1902107 | positive regulation of leukocyte differentiation | 1.63E-09 |
| BP | GO:1903708 | positive regulation of hemopoiesis | 1.63E-09 |
| BP | GO:0001912 | positive regulation of leukocyte mediated cytotoxicity | 1.71E-09 |
| BP | GO:0019882 | antigen processing and presentation | 1.76E-09 |
| BP | GO:0043383 | negative T cell selection | 1.77E-09 |
| BP | GO:0042130 | negative regulation of T cell proliferation | 2.04E-09 |
| BP | GO:0002287 | alpha-beta T cell activation involved in immune response | 2.51E-09 |
| BP | GO:0002293 | alpha-beta T cell differentiation involved in immune response | 2.51E-09 |
| BP | GO:0046637 | regulation of alpha-beta T cell differentiation | 2.51E-09 |
| BP | GO:0007204 | positive regulation of cytosolic calcium ion concentration | 2.64E-09 |
| BP | GO:0072676 | lymphocyte migration | 3.72E-09 |
| BP | GO:0051607 | defense response to virus | 3.74E-09 |
| BP | GO:0140546 | defense response to symbiont | 3.74E-09 |
| BP | GO:0032663 | regulation of interleukin-2 production | 4.40E-09 |
| BP | GO:0032615 | interleukin-12 production | 5.53E-09 |
| BP | GO:0032655 | regulation of interleukin-12 production | 5.53E-09 |
| BP | GO:0032623 | interleukin-2 production | 6.96E-09 |
| BP | GO:0051480 | regulation of cytosolic calcium ion concentration | 7.98E-09 |
| BP | GO:0060326 | cell chemotaxis | 8.03E-09 |
| BP | GO:0045954 | positive regulation of natural killer cell mediated cytotoxicity | 8.33E-09 |
| BP | GO:0032613 | interleukin-10 production | 8.55E-09 |
| BP | GO:0043373 | CD4-positive, alpha-beta T cell lineage commitment | 1.15E-08 |
| BP | GO:0002704 | negative regulation of leukocyte mediated immunity | 1.18E-08 |
| BP | GO:0030595 | leukocyte chemotaxis | 1.89E-08 |
| BP | GO:0002363 | alpha-beta T cell lineage commitment | 1.94E-08 |
| BP | GO:0002294 | CD4-positive, alpha-beta T cell differentiation involved in immune response | 2.00E-08 |
| BP | GO:0002700 | regulation of production of molecular mediator of immune response | 2.14E-08 |
| BP | GO:0006874 | cellular calcium ion homeostasis | 2.33E-08 |
| BP | GO:0002821 | positive regulation of adaptive immune response | 2.36E-08 |
| BP | GO:0043369 | CD4-positive or CD8-positive, alpha-beta T cell lineage commitment | 3.08E-08 |
| BP | GO:0045061 | thymic T cell selection | 3.08E-08 |
| BP | GO:0050777 | negative regulation of immune response | 3.37E-08 |
| BP | GO:0045060 | negative thymic T cell selection | 3.85E-08 |
| BP | GO:0055074 | calcium ion homeostasis | 4.59E-08 |
| BP | GO:1990266 | neutrophil migration | 4.71E-08 |
| BP | GO:0046638 | positive regulation of alpha-beta T cell differentiation | 5.08E-08 |
| BP | GO:0042100 | B cell proliferation | 5.15E-08 |
| BP | GO:0032653 | regulation of interleukin-10 production | 5.58E-08 |
| BP | GO:0002718 | regulation of cytokine production involved in immune response | 5.92E-08 |
| BP | GO:0002295 | T-helper cell lineage commitment | 6.17E-08 |
| BP | GO:0022408 | negative regulation of cell-cell adhesion | 6.69E-08 |
| BP | GO:0043372 | positive regulation of CD4-positive, alpha-beta T cell differentiation | 7.68E-08 |
| BP | GO:0002824 | positive regulation of adaptive immune response based on somatic recombination of immune receptors built from immunoglobulin superfamily domains | 7.90E-08 |
| BP | GO:0043370 | regulation of CD4-positive, alpha-beta T cell differentiation | 7.96E-08 |
| BP | GO:0002643 | regulation of tolerance induction | 1.05E-07 |
| BP | GO:0045589 | regulation of regulatory T cell differentiation | 1.05E-07 |
| BP | GO:0042093 | T-helper cell differentiation | 1.23E-07 |
| BP | GO:0097530 | granulocyte migration | 1.47E-07 |
| BP | GO:0019722 | calcium-mediated signaling | 1.52E-07 |
| BP | GO:0072503 | cellular divalent inorganic cation homeostasis | 1.81E-07 |
| BP | GO:0097529 | myeloid leukocyte migration | 1.88E-07 |
| BP | GO:2000514 | regulation of CD4-positive, alpha-beta T cell activation | 2.14E-07 |
| BP | GO:0002360 | T cell lineage commitment | 2.28E-07 |
| BP | GO:0002367 | cytokine production involved in immune response | 2.32E-07 |
| BP | GO:0002517 | T cell tolerance induction | 3.26E-07 |
| BP | GO:0045059 | positive thymic T cell selection | 3.26E-07 |
| BP | GO:0072540 | T-helper 17 cell lineage commitment | 3.26E-07 |
| BP | GO:0097028 | dendritic cell differentiation | 3.45E-07 |
| BP | GO:0002698 | negative regulation of immune effector process | 3.51E-07 |
| BP | GO:0001773 | myeloid dendritic cell activation | 4.46E-07 |
| BP | GO:0032496 | response to lipopolysaccharide | 4.46E-07 |
| BP | GO:0072507 | divalent inorganic cation homeostasis | 4.59E-07 |
| BP | GO:0019886 | antigen processing and presentation of exogenous peptide antigen via MHC class II | 5.28E-07 |
| BP | GO:2000516 | positive regulation of CD4-positive, alpha-beta T cell activation | 5.71E-07 |
| BP | GO:0030888 | regulation of B cell proliferation | 6.17E-07 |
| BP | GO:0050856 | regulation of T cell receptor signaling pathway | 7.36E-07 |
| BP | GO:0002291 | T cell activation via T cell receptor contact with antigen bound to MHC molecule on antigen presenting cell | 7.96E-07 |
| BP | GO:0007162 | negative regulation of cell adhesion | 8.05E-07 |
| BP | GO:0002687 | positive regulation of leukocyte migration | 8.51E-07 |
| BP | GO:0002495 | antigen processing and presentation of peptide antigen via MHC class II | 8.75E-07 |
| BP | GO:0030593 | neutrophil chemotaxis | 8.75E-07 |
| BP | GO:0050857 | positive regulation of antigen receptor-mediated signaling pathway | 9.14E-07 |
| BP | GO:0002504 | antigen processing and presentation of peptide or polysaccharide antigen via MHC class II | 9.89E-07 |
| BP | GO:0048247 | lymphocyte chemotaxis | 1.04E-06 |
| BP | GO:0002690 | positive regulation of leukocyte chemotaxis | 1.17E-06 |
| BP | GO:0002685 | regulation of leukocyte migration | 1.19E-06 |
| BP | GO:0002407 | dendritic cell chemotaxis | 1.31E-06 |
| BP | GO:0032633 | interleukin-4 production | 1.44E-06 |
| BP | GO:0002237 | response to molecule of bacterial origin | 1.44E-06 |
| BP | GO:0050921 | positive regulation of chemotaxis | 1.55E-06 |
| BP | GO:0002645 | positive regulation of tolerance induction | 1.60E-06 |
| BP | GO:0071621 | granulocyte chemotaxis | 1.71E-06 |
| BP | GO:0048002 | antigen processing and presentation of peptide antigen | 2.10E-06 |
| BP | GO:0002707 | negative regulation of lymphocyte mediated immunity | 2.25E-06 |
| BP | GO:0002478 | antigen processing and presentation of exogenous peptide antigen | 2.25E-06 |
| BP | GO:0002664 | regulation of T cell tolerance induction | 3.06E-06 |
| BP | GO:0036037 | CD8-positive, alpha-beta T cell activation | 3.52E-06 |
| BP | GO:0046633 | alpha-beta T cell proliferation | 4.03E-06 |
| BP | GO:0001913 | T cell mediated cytotoxicity | 4.22E-06 |
| BP | GO:0050727 | regulation of inflammatory response | 4.35E-06 |
| BP | GO:0019884 | antigen processing and presentation of exogenous antigen | 4.39E-06 |
| BP | GO:0071887 | leukocyte apoptotic process | 4.63E-06 |
| BP | GO:0001771 | immunological synapse formation | 5.38E-06 |
| BP | GO:0002577 | regulation of antigen processing and presentation | 5.38E-06 |
| BP | GO:0002688 | regulation of leukocyte chemotaxis | 5.43E-06 |
| BP | GO:0036336 | dendritic cell migration | 6.21E-06 |
| BP | GO:0072539 | T-helper 17 cell differentiation | 6.21E-06 |
| BP | GO:0045622 | regulation of T-helper cell differentiation | 6.27E-06 |
| BP | GO:0046641 | positive regulation of alpha-beta T cell proliferation | 7.72E-06 |
| BP | GO:0032735 | positive regulation of interleukin-12 production | 7.84E-06 |
| BP | GO:0032743 | positive regulation of interleukin-2 production | 8.13E-06 |
| BP | GO:0042088 | T-helper 1 type immune response | 9.74E-06 |
| BP | GO:0002710 | negative regulation of T cell mediated immunity | 1.09E-05 |
| BP | GO:0071222 | cellular response to lipopolysaccharide | 1.09E-05 |
| BP | GO:2000106 | regulation of leukocyte apoptotic process | 1.13E-05 |
| BP | GO:0032673 | regulation of interleukin-4 production | 1.37E-05 |
| BP | GO:0002468 | dendritic cell antigen processing and presentation | 1.41E-05 |
| BP | GO:0046006 | regulation of activated T cell proliferation | 1.46E-05 |
| BP | GO:0002548 | monocyte chemotaxis | 1.47E-05 |
| BP | GO:0032693 | negative regulation of interleukin-10 production | 1.48E-05 |
| BP | GO:0072538 | T-helper 17 type immune response | 1.73E-05 |
| BP | GO:0030101 | natural killer cell activation | 1.88E-05 |
| BP | GO:0002418 | immune response to tumor cell | 2.02E-05 |
| BP | GO:0032753 | positive regulation of interleukin-4 production | 2.02E-05 |
| BP | GO:0002260 | lymphocyte homeostasis | 2.02E-05 |
| BP | GO:0045591 | positive regulation of regulatory T cell differentiation | 2.13E-05 |
| BP | GO:0046640 | regulation of alpha-beta T cell proliferation | 2.16E-05 |
| BP | GO:0070227 | lymphocyte apoptotic process | 2.20E-05 |
| BP | GO:0071219 | cellular response to molecule of bacterial origin | 2.50E-05 |
| BP | GO:0050798 | activated T cell proliferation | 2.53E-05 |
| BP | GO:0033077 | T cell differentiation in thymus | 2.88E-05 |
| BP | GO:0031348 | negative regulation of defense response | 3.97E-05 |
| BP | GO:0001818 | negative regulation of cytokine production | 4.14E-05 |
| BP | GO:0002834 | regulation of response to tumor cell | 4.54E-05 |
| BP | GO:0002837 | regulation of immune response to tumor cell | 4.54E-05 |
| BP | GO:0032695 | negative regulation of interleukin-12 production | 4.54E-05 |
| BP | GO:0010818 | T cell chemotaxis | 4.59E-05 |
| BP | GO:0071677 | positive regulation of mononuclear cell migration | 4.95E-05 |
| BP | GO:0033632 | regulation of cell-cell adhesion mediated by integrin | 5.01E-05 |
| BP | GO:0001914 | regulation of T cell mediated cytotoxicity | 5.04E-05 |
| BP | GO:0032703 | negative regulation of interleukin-2 production | 5.89E-05 |
| BP | GO:0001776 | leukocyte homeostasis | 6.10E-05 |
| BP | GO:0071675 | regulation of mononuclear cell migration | 6.26E-05 |
| BP | GO:0002544 | chronic inflammatory response | 6.27E-05 |
| BP | GO:0002716 | negative regulation of natural killer cell mediated immunity | 6.27E-05 |
| BP | GO:0060337 | type I interferon signaling pathway | 6.41E-05 |
| BP | GO:0071357 | cellular response to type I interferon | 7.13E-05 |
| BP | GO:1903900 | regulation of viral life cycle | 7.31E-05 |
| BP | GO:0072678 | T cell migration | 7.32E-05 |
| BP | GO:0034154 | toll-like receptor 7 signaling pathway | 8.03E-05 |
| BP | GO:0043380 | regulation of memory T cell differentiation | 8.03E-05 |
| BP | GO:0050848 | regulation of calcium-mediated signaling | 8.31E-05 |
| BP | GO:0032689 | negative regulation of interferon-gamma production | 8.76E-05 |
| BP | GO:0070228 | regulation of lymphocyte apoptotic process | 9.29E-05 |
| BP | GO:0002702 | positive regulation of production of molecular mediator of immune response | 9.55E-05 |
| BP | GO:1902622 | regulation of neutrophil migration | 0.000105 |
| BP | GO:0071216 | cellular response to biotic stimulus | 0.00011 |
| BP | GO:0033630 | positive regulation of cell adhesion mediated by integrin | 0.000114 |
| BP | GO:0002347 | response to tumor cell | 0.000114 |
| BP | GO:0034340 | response to type I interferon | 0.000115 |
| BP | GO:0043379 | memory T cell differentiation | 0.000123 |
| BP | GO:0002861 | regulation of inflammatory response to antigenic stimulus | 0.000142 |
| BP | GO:0001911 | negative regulation of leukocyte mediated cytotoxicity | 0.000151 |
| BP | GO:0019932 | second-messenger-mediated signaling | 0.000172 |
| BP | GO:0002724 | regulation of T cell cytokine production | 0.000174 |
| BP | GO:0035589 | G protein-coupled purinergic nucleotide receptor signaling pathway | 0.000183 |
| BP | GO:0050862 | positive regulation of T cell receptor signaling pathway | 0.000183 |
| BP | GO:0090715 | immunological memory formation process | 0.000183 |
| BP | GO:2000107 | negative regulation of leukocyte apoptotic process | 0.000202 |
| BP | GO:0045576 | mast cell activation | 0.000213 |
| BP | GO:0050920 | regulation of chemotaxis | 0.000216 |
| BP | GO:0002823 | negative regulation of adaptive immune response based on somatic recombination of immune receptors built from immunoglobulin superfamily domains | 0.000235 |
| BP | GO:0002836 | positive regulation of response to tumor cell | 0.000262 |
| BP | GO:0002839 | positive regulation of immune response to tumor cell | 0.000262 |
| BP | GO:0046007 | negative regulation of activated T cell proliferation | 0.000262 |
| BP | GO:0033628 | regulation of cell adhesion mediated by integrin | 0.000272 |
| BP | GO:0002437 | inflammatory response to antigenic stimulus | 0.000272 |
| BP | GO:0002218 | activation of innate immune response | 0.000289 |
| BP | GO:0071356 | cellular response to tumor necrosis factor | 0.000293 |
| BP | GO:0002369 | T cell cytokine production | 0.000302 |
| BP | GO:0034612 | response to tumor necrosis factor | 0.000306 |
| BP | GO:0070231 | T cell apoptotic process | 0.000312 |
| BP | GO:0001562 | response to protozoan | 0.000312 |
| BP | GO:0033622 | integrin activation | 0.000312 |
| BP | GO:0042832 | defense response to protozoan | 0.000312 |
| BP | GO:2000403 | positive regulation of lymphocyte migration | 0.000357 |
| BP | GO:0050729 | positive regulation of inflammatory response | 0.000381 |
| BP | GO:0002719 | negative regulation of cytokine production involved in immune response | 0.000389 |
| BP | GO:0031342 | negative regulation of cell killing | 0.000389 |
| BP | GO:0034162 | toll-like receptor 9 signaling pathway | 0.000389 |
| BP | GO:1901623 | regulation of lymphocyte chemotaxis | 0.000389 |
| BP | GO:0032733 | positive regulation of interleukin-10 production | 0.00042 |
| BP | GO:0002711 | positive regulation of T cell mediated immunity | 0.000468 |
| BP | GO:0002820 | negative regulation of adaptive immune response | 0.000468 |
| BP | GO:0002825 | regulation of T-helper 1 type immune response | 0.00048 |
| BP | GO:0090025 | regulation of monocyte chemotaxis | 0.00048 |
| BP | GO:0033631 | cell-cell adhesion mediated by integrin | 0.00048 |
| BP | GO:0090713 | immunological memory process | 0.00048 |
| BP | GO:0002701 | negative regulation of production of molecular mediator of immune response | 0.00049 |
| BP | GO:0030183 | B cell differentiation | 0.000498 |
| BP | GO:0002720 | positive regulation of cytokine production involved in immune response | 0.000527 |
| BP | GO:0002381 | immunoglobulin production involved in immunoglobulin-mediated immune response | 0.000601 |
| BP | GO:0045071 | negative regulation of viral genome replication | 0.000601 |
| BP | GO:0045953 | negative regulation of natural killer cell mediated cytotoxicity | 0.000639 |
| BP | GO:0002765 | immune response-inhibiting signal transduction | 0.000712 |
| BP | GO:0050690 | regulation of defense response to virus by virus | 0.000717 |
| BP | GO:0002573 | myeloid leukocyte differentiation | 0.000726 |
| BP | GO:1903555 | regulation of tumor necrosis factor superfamily cytokine production | 0.000811 |
| BP | GO:0046629 | gamma-delta T cell activation | 0.000833 |
| BP | GO:2001185 | regulation of CD8-positive, alpha-beta T cell activation | 0.000833 |
| BP | GO:0045577 | regulation of B cell differentiation | 0.000861 |
| BP | GO:0050858 | negative regulation of antigen receptor-mediated signaling pathway | 0.000861 |
| BP | GO:0050792 | regulation of viral process | 0.000865 |
| BP | GO:0019058 | viral life cycle | 0.000954 |
| BP | GO:0071706 | tumor necrosis factor superfamily cytokine production | 0.000964 |
| BP | GO:0019835 | cytolysis | 0.001032 |
| BP | GO:0002604 | regulation of dendritic cell antigen processing and presentation | 0.001044 |
| BP | GO:0033625 | positive regulation of integrin activation | 0.001044 |
| BP | GO:0035747 | natural killer cell chemotaxis | 0.001044 |
| BP | GO:0097048 | dendritic cell apoptotic process | 0.001044 |
| BP | GO:2000668 | regulation of dendritic cell apoptotic process | 0.001044 |
| BP | GO:0090026 | positive regulation of monocyte chemotaxis | 0.001046 |
| BP | GO:0140131 | positive regulation of lymphocyte chemotaxis | 0.001046 |
| BP | GO:0019079 | viral genome replication | 0.001076 |
| BP | GO:0051209 | release of sequestered calcium ion into cytosol | 0.001254 |
| BP | GO:0060402 | calcium ion transport into cytosol | 0.001331 |
| BP | GO:0051283 | negative regulation of sequestering of calcium ion | 0.00135 |
| BP | GO:0006816 | calcium ion transport | 0.001419 |
| BP | GO:0050869 | negative regulation of B cell activation | 0.001427 |
| BP | GO:0051482 | positive regulation of cytosolic calcium ion concentration involved in phospholipase C-activating G protein-coupled signaling pathway | 0.001427 |
| BP | GO:0001768 | establishment of T cell polarity | 0.00149 |
| BP | GO:0045581 | negative regulation of T cell differentiation | 0.00152 |
| BP | GO:0043903 | regulation of biological process involved in symbiotic interaction | 0.001543 |
| BP | GO:0051282 | regulation of sequestering of calcium ion | 0.001547 |
| BP | GO:0050860 | negative regulation of T cell receptor signaling pathway | 0.001645 |
| BP | GO:0070232 | regulation of T cell apoptotic process | 0.001666 |
| BP | GO:0071622 | regulation of granulocyte chemotaxis | 0.001958 |
| BP | GO:0045824 | negative regulation of innate immune response | 0.002039 |
| BP | GO:0002832 | negative regulation of response to biotic stimulus | 0.002039 |
| BP | GO:0051208 | sequestering of calcium ion | 0.002039 |
| BP | GO:0001767 | establishment of lymphocyte polarity | 0.002039 |
| BP | GO:0048302 | regulation of isotype switching to IgG isotypes | 0.002039 |
| BP | GO:0070234 | positive regulation of T cell apoptotic process | 0.002039 |
| BP | GO:0032680 | regulation of tumor necrosis factor production | 0.002103 |
| BP | GO:0045069 | regulation of viral genome replication | 0.002226 |
| BP | GO:0042092 | type 2 immune response | 0.002235 |
| BP | GO:0043029 | T cell homeostasis | 0.002235 |
| BP | GO:0045730 | respiratory burst | 0.002235 |
| BP | GO:0032640 | tumor necrosis factor production | 0.002342 |
| BP | GO:0090023 | positive regulation of neutrophil chemotaxis | 0.00244 |
| BP | GO:0002637 | regulation of immunoglobulin production | 0.00244 |
| BP | GO:0050766 | positive regulation of phagocytosis | 0.00244 |
| BP | GO:0019730 | antimicrobial humoral response | 0.002442 |
| BP | GO:0043374 | CD8-positive, alpha-beta T cell differentiation | 0.002707 |
| BP | GO:0048291 | isotype switching to IgG isotypes | 0.002707 |
| BP | GO:0070374 | positive regulation of ERK1 and ERK2 cascade | 0.002884 |
| BP | GO:0050688 | regulation of defense response to virus | 0.002951 |
| BP | GO:0032760 | positive regulation of tumor necrosis factor production | 0.003064 |
| BP | GO:0007249 | I-kappaB kinase/NF-kappaB signaling | 0.003084 |
| BP | GO:0045123 | cellular extravasation | 0.003232 |
| BP | GO:0032660 | regulation of interleukin-17 production | 0.003387 |
| BP | GO:2000108 | positive regulation of leukocyte apoptotic process | 0.003508 |
| BP | GO:0048525 | negative regulation of viral process | 0.003581 |
| BP | GO:0045620 | negative regulation of lymphocyte differentiation | 0.003764 |
| BP | GO:0030890 | positive regulation of B cell proliferation | 0.003849 |
| BP | GO:0033627 | cell adhesion mediated by integrin | 0.003849 |
| BP | GO:1903557 | positive regulation of tumor necrosis factor superfamily cytokine production | 0.003849 |
| BP | GO:0071624 | positive regulation of granulocyte chemotaxis | 0.004149 |
| BP | GO:0060401 | cytosolic calcium ion transport | 0.004174 |
| BP | GO:0010819 | regulation of T cell chemotaxis | 0.004531 |
| BP | GO:0070230 | positive regulation of lymphocyte apoptotic process | 0.004531 |
| BP | GO:0001782 | B cell homeostasis | 0.004857 |
| BP | GO:0032607 | interferon-alpha production | 0.004857 |
| BP | GO:0032647 | regulation of interferon-alpha production | 0.004857 |
| BP | GO:0050855 | regulation of B cell receptor signaling pathway | 0.004857 |
| BP | GO:0002763 | positive regulation of myeloid leukocyte differentiation | 0.00506 |
| BP | GO:0097553 | calcium ion transmembrane import into cytosol | 0.005146 |
| BP | GO:0002223 | stimulatory C-type lectin receptor signaling pathway | 0.005305 |
| BP | GO:0032620 | interleukin-17 production | 0.005539 |
| BP | GO:0048872 | homeostasis of number of cells | 0.005601 |
| BP | GO:0002224 | toll-like receptor signaling pathway | 0.005605 |
| BP | GO:0001916 | positive regulation of T cell mediated cytotoxicity | 0.005605 |
| BP | GO:1902624 | positive regulation of neutrophil migration | 0.005605 |
| BP | GO:0030889 | negative regulation of B cell proliferation | 0.005605 |
| BP | GO:0033623 | regulation of integrin activation | 0.005605 |
| BP | GO:0043123 | positive regulation of I-kappaB kinase/NF-kappaB signaling | 0.006423 |
| BP | GO:0050728 | negative regulation of inflammatory response | 0.006488 |
| BP | GO:0002828 | regulation of type 2 immune response | 0.006488 |
| BP | GO:0050850 | positive regulation of calcium-mediated signaling | 0.006488 |
| BP | GO:0050901 | leukocyte tethering or rolling | 0.006488 |
| BP | GO:2000406 | positive regulation of T cell migration | 0.006488 |
| BP | GO:0002220 | innate immune response activating cell surface receptor signaling pathway | 0.006682 |
| BP | GO:0002761 | regulation of myeloid leukocyte differentiation | 0.006682 |
| BP | GO:0070269 | pyroptosis | 0.006906 |
| BP | GO:0046718 | viral entry into host cell | 0.007075 |
| BP | GO:0002758 | innate immune response-activating signal transduction | 0.007079 |
| BP | GO:0070229 | negative regulation of lymphocyte apoptotic process | 0.007464 |
| BP | GO:2000401 | regulation of lymphocyte migration | 0.007899 |
| BP | GO:1902106 | negative regulation of leukocyte differentiation | 0.008197 |
| BP | GO:0048245 | eosinophil chemotaxis | 0.008428 |
| BP | GO:2000319 | regulation of T-helper 17 cell differentiation | 0.008428 |
| BP | GO:0001774 | microglial cell activation | 0.008457 |
| BP | GO:0035590 | purinergic nucleotide receptor signaling pathway | 0.008518 |
| BP | GO:0090022 | regulation of neutrophil chemotaxis | 0.008518 |
| BP | GO:0032732 | positive regulation of interleukin-1 production | 0.008532 |
| BP | GO:0042116 | macrophage activation | 0.008625 |
| BP | GO:0045639 | positive regulation of myeloid cell differentiation | 0.008625 |
| BP | GO:0042119 | neutrophil activation | 0.008907 |
| BP | GO:0051924 | regulation of calcium ion transport | 0.009136 |
| BP | GO:0007200 | phospholipase C-activating G protein-coupled receptor signaling pathway | 0.00917 |
| BP | GO:0030099 | myeloid cell differentiation | 0.00917 |
| BP | GO:0002204 | somatic recombination of immunoglobulin genes involved in immune response | 0.009197 |
| BP | GO:0002208 | somatic diversification of immunoglobulins involved in immune response | 0.009197 |
| BP | GO:0045190 | isotype switching | 0.009197 |
| BP | GO:0032814 | regulation of natural killer cell activation | 0.009536 |
| BP | GO:0035025 | positive regulation of Rho protein signal transduction | 0.009536 |
| BP | GO:0043552 | positive regulation of phosphatidylinositol 3-kinase activity | 0.009536 |
| BP | GO:0032102 | negative regulation of response to external stimulus | 0.009569 |
| BP | GO:0043011 | myeloid dendritic cell differentiation | 0.009877 |
| BP | GO:0070233 | negative regulation of T cell apoptotic process | 0.009877 |
| BP | GO:1903707 | negative regulation of hemopoiesis | 0.01024 |
| BP | GO:0032612 | interleukin-1 production | 0.010288 |
| BP | GO:0043122 | regulation of I-kappaB kinase/NF-kappaB signaling | 0.011302 |
| BP | GO:0002725 | negative regulation of T cell cytokine production | 0.011464 |
| BP | GO:0035455 | response to interferon-alpha | 0.01182 |
| BP | GO:0033209 | tumor necrosis factor-mediated signaling pathway | 0.012232 |
| BP | GO:0032611 | interleukin-1 beta production | 0.012289 |
| BP | GO:0030001 | metal ion transport | 0.012408 |
| BP | GO:0010959 | regulation of metal ion transport | 0.012558 |
| BP | GO:0044409 | entry into host | 0.013325 |
| BP | GO:0032700 | negative regulation of interleukin-17 production | 0.013905 |
| BP | GO:0045624 | positive regulation of T-helper cell differentiation | 0.013905 |
| BP | GO:2000316 | regulation of T-helper 17 type immune response | 0.013905 |
| BP | GO:0070372 | regulation of ERK1 and ERK2 cascade | 0.013931 |
| BP | GO:1905517 | macrophage migration | 0.014727 |
| BP | GO:0034163 | regulation of toll-like receptor 9 signaling pathway | 0.014827 |
| BP | GO:0035457 | cellular response to interferon-alpha | 0.014827 |
| BP | GO:0045657 | positive regulation of monocyte differentiation | 0.014827 |
| BP | GO:0046598 | positive regulation of viral entry into host cell | 0.014827 |
| BP | GO:0046643 | regulation of gamma-delta T cell activation | 0.014827 |
| BP | GO:0052697 | xenobiotic glucuronidation | 0.014827 |
| BP | GO:0075294 | positive regulation by symbiont of entry into host | 0.014827 |
| BP | GO:0098883 | synapse pruning | 0.014827 |
| BP | GO:0090218 | positive regulation of lipid kinase activity | 0.015158 |
| BP | GO:0002712 | regulation of B cell mediated immunity | 0.015797 |
| BP | GO:0002889 | regulation of immunoglobulin mediated immune response | 0.015797 |
| BP | GO:0016447 | somatic recombination of immunoglobulin gene segments | 0.015797 |
| BP | GO:0002438 | acute inflammatory response to antigenic stimulus | 0.01593 |
| BP | GO:0072677 | eosinophil migration | 0.01593 |
| BP | GO:0032675 | regulation of interleukin-6 production | 0.016404 |
| BP | GO:0002714 | positive regulation of B cell mediated immunity | 0.0168 |
| BP | GO:0002891 | positive regulation of immunoglobulin mediated immune response | 0.0168 |
| BP | GO:0050764 | regulation of phagocytosis | 0.017903 |
| BP | GO:0002221 | pattern recognition receptor signaling pathway | 0.018095 |
| BP | GO:1905523 | positive regulation of macrophage migration | 0.018494 |
| BP | GO:0032731 | positive regulation of interleukin-1 beta production | 0.01866 |
| BP | GO:1905521 | regulation of macrophage migration | 0.018667 |
| BP | GO:0002863 | positive regulation of inflammatory response to antigenic stimulus | 0.018667 |
| BP | GO:0033004 | negative regulation of mast cell activation | 0.018667 |
| BP | GO:0038110 | interleukin-2-mediated signaling pathway | 0.018667 |
| BP | GO:0042492 | gamma-delta T cell differentiation | 0.018667 |
| BP | GO:0032635 | interleukin-6 production | 0.019394 |
| BP | GO:0043551 | regulation of phosphatidylinositol 3-kinase activity | 0.020115 |
| BP | GO:0035306 | positive regulation of dephosphorylation | 0.021867 |
| BP | GO:0032652 | regulation of interleukin-1 production | 0.021867 |
| BP | GO:0033003 | regulation of mast cell activation | 0.022775 |
| BP | GO:0046596 | regulation of viral entry into host cell | 0.022775 |
| BP | GO:0050691 | regulation of defense response to virus by host | 0.022775 |
| BP | GO:0043312 | neutrophil degranulation | 0.023029 |
| BP | GO:0002430 | complement receptor mediated signaling pathway | 0.023226 |
| BP | GO:0035723 | interleukin-15-mediated signaling pathway | 0.023226 |
| BP | GO:0071350 | cellular response to interleukin-15 | 0.023226 |
| BP | GO:0071352 | cellular response to interleukin-2 | 0.023226 |
| BP | GO:0070371 | ERK1 and ERK2 cascade | 0.023895 |
| BP | GO:0042104 | positive regulation of activated T cell proliferation | 0.023973 |
| BP | GO:0002283 | neutrophil activation involved in immune response | 0.024453 |
| BP | GO:0002312 | B cell activation involved in immune response | 0.024478 |
| BP | GO:0034113 | heterotypic cell-cell adhesion | 0.025229 |
| BP | GO:0009988 | cell-cell recognition | 0.026139 |
| BP | GO:0010758 | regulation of macrophage chemotaxis | 0.027301 |
| BP | GO:2000404 | regulation of T cell migration | 0.027321 |
| BP | GO:0002864 | regulation of acute inflammatory response to antigenic stimulus | 0.028366 |
| BP | GO:0070669 | response to interleukin-2 | 0.028366 |
| BP | GO:0070672 | response to interleukin-15 | 0.028366 |
| BP | GO:0032651 | regulation of interleukin-1 beta production | 0.029324 |
| BP | GO:0002639 | positive regulation of immunoglobulin production | 0.029885 |
| BP | GO:0002446 | neutrophil mediated immunity | 0.03117 |
| BP | GO:0051897 | positive regulation of protein kinase B signaling | 0.032775 |
| BP | GO:0052126 | movement in host environment | 0.032775 |
| BP | GO:0032479 | regulation of type I interferon production | 0.033492 |
| BP | GO:0002562 | somatic diversification of immune receptors via germline recombination within a single locus | 0.033654 |
| BP | GO:0016444 | somatic cell DNA recombination | 0.033654 |
| BP | GO:0016445 | somatic diversification of immunoglobulins | 0.033654 |
| BP | GO:0002827 | positive regulation of T-helper 1 type immune response | 0.03391 |
| BP | GO:0009812 | flavonoid metabolic process | 0.03391 |
| BP | GO:0010820 | positive regulation of T cell chemotaxis | 0.03391 |
| BP | GO:0032645 | regulation of granulocyte macrophage colony-stimulating factor production | 0.03391 |
| BP | GO:0032606 | type I interferon production | 0.034663 |
| BP | GO:0007157 | heterophilic cell-cell adhesion via plasma membrane cell adhesion molecules | 0.038569 |
| BP | GO:0035722 | interleukin-12-mediated signaling pathway | 0.038569 |
| BP | GO:0045637 | regulation of myeloid cell differentiation | 0.040321 |
| BP | GO:0002830 | positive regulation of type 2 immune response | 0.040321 |
| BP | GO:0032604 | granulocyte macrophage colony-stimulating factor production | 0.040321 |
| BP | GO:0044764 | multi-organism cellular process | 0.040321 |
| BP | GO:0060263 | regulation of respiratory burst | 0.040321 |
| BP | GO:0052372 | modulation by symbiont of entry into host | 0.041681 |
| BP | GO:0002230 | positive regulation of defense response to virus by host | 0.042664 |
| BP | GO:0002323 | natural killer cell activation involved in immune response | 0.042664 |
| BP | GO:0035456 | response to interferon-beta | 0.042664 |
| BP | GO:0051651 | maintenance of location in cell | 0.044465 |
| BP | GO:0071349 | cellular response to interleukin-12 | 0.045028 |
| BP | GO:0043550 | regulation of lipid kinase activity | 0.046609 |
| BP | GO:0010759 | positive regulation of macrophage chemotaxis | 0.046985 |
| BP | GO:0048535 | lymph node development | 0.046985 |
| BP | GO:0150079 | negative regulation of neuroinflammatory response | 0.046985 |
| BP | GO:0010453 | regulation of cell fate commitment | 0.046985 |
| BP | GO:0071353 | cellular response to interleukin-4 | 0.046985 |
| BP | GO:1903902 | positive regulation of viral life cycle | 0.046985 |
| BP | GO:0070671 | response to interleukin-12 | 0.048268 |
| BP | GO:0048524 | positive regulation of viral process | 0.048392 |
| CC | GO:0019814 | immunoglobulin complex | 2.08E-122 |
| CC | GO:0009897 | external side of plasma membrane | 1.17E-75 |
| CC | GO:0042101 | T cell receptor complex | 9.22E-69 |
| CC | GO:0042571 | immunoglobulin complex, circulating | 4.36E-61 |
| CC | GO:0098802 | plasma membrane signaling receptor complex | 3.85E-50 |
| CC | GO:0042613 | MHC class II protein complex | 4.01E-17 |
| CC | GO:0001772 | immunological synapse | 2.17E-15 |
| CC | GO:0072562 | blood microparticle | 2.02E-14 |
| CC | GO:0042611 | MHC protein complex | 2.01E-13 |
| CC | GO:0030669 | clathrin-coated endocytic vesicle membrane | 3.84E-09 |
| CC | GO:0071556 | integral component of lumenal side of endoplasmic reticulum membrane | 3.10E-08 |
| CC | GO:0098553 | lumenal side of endoplasmic reticulum membrane | 3.10E-08 |
| CC | GO:0045334 | clathrin-coated endocytic vesicle | 3.10E-08 |
| CC | GO:0098576 | lumenal side of membrane | 2.92E-07 |
| CC | GO:0030665 | clathrin-coated vesicle membrane | 5.48E-06 |
| CC | GO:0012507 | ER to Golgi transport vesicle membrane | 5.95E-05 |
| CC | GO:0030139 | endocytic vesicle | 0.000237 |
| CC | GO:0030666 | endocytic vesicle membrane | 0.000296 |
| CC | GO:0030136 | clathrin-coated vesicle | 0.000512 |
| CC | GO:0030662 | coated vesicle membrane | 0.000959 |
| CC | GO:0030134 | COPII-coated ER to Golgi transport vesicle | 0.001836 |
| CC | GO:0032588 | trans-Golgi network membrane | 0.001915 |
| CC | GO:0045121 | membrane raft | 0.002965 |
| CC | GO:0098857 | membrane microdomain | 0.002965 |
| CC | GO:0070821 | tertiary granule membrane | 0.027678 |
| CC | GO:0070820 | tertiary granule | 0.034507 |
| CC | GO:0001931 | uropod | 0.036002 |
| CC | GO:0031254 | cell trailing edge | 0.036002 |
| CC | GO:0030135 | coated vesicle | 0.036002 |
| CC | GO:0005765 | lysosomal membrane | 0.036002 |
| CC | GO:0098852 | lytic vacuole membrane | 0.036002 |
| CC | GO:0101003 | ficolin-1-rich granule membrane | 0.038018 |
| CC | GO:0002102 | podosome | 0.042614 |
| CC | GO:0030667 | secretory granule membrane | 0.043284 |
| MF | GO:0003823 | antigen binding | 1.96E-108 |
| MF | GO:0034987 | immunoglobulin receptor binding | 1.18E-60 |
| MF | GO:0140375 | immune receptor activity | 2.26E-31 |
| MF | GO:0004896 | cytokine receptor activity | 2.07E-15 |
| MF | GO:0023023 | MHC protein complex binding | 4.78E-15 |
| MF | GO:0019955 | cytokine binding | 3.45E-12 |
| MF | GO:0042605 | peptide antigen binding | 1.24E-10 |
| MF | GO:0042287 | MHC protein binding | 1.24E-10 |
| MF | GO:0032395 | MHC class II receptor activity | 1.30E-10 |
| MF | GO:0001637 | G protein-coupled chemoattractant receptor activity | 4.67E-09 |
| MF | GO:0004950 | chemokine receptor activity | 4.67E-09 |
| MF | GO:0016493 | C-C chemokine receptor activity | 3.09E-08 |
| MF | GO:0023026 | MHC class II protein complex binding | 4.16E-08 |
| MF | GO:0019957 | C-C chemokine binding | 4.16E-08 |
| MF | GO:0015026 | coreceptor activity | 2.10E-07 |
| MF | GO:0019956 | chemokine binding | 8.95E-07 |
| MF | GO:0030246 | carbohydrate binding | 1.18E-06 |
| MF | GO:0042608 | T cell receptor binding | 1.26E-06 |
| MF | GO:0008009 | chemokine activity | 2.62E-06 |
| MF | GO:0042379 | chemokine receptor binding | 8.27E-06 |
| MF | GO:0005126 | cytokine receptor binding | 1.99E-05 |
| MF | GO:0005125 | cytokine activity | 3.51E-05 |
| MF | GO:0048020 | CCR chemokine receptor binding | 0.000144 |
| MF | GO:0045028 | G protein-coupled purinergic nucleotide receptor activity | 0.00017 |
| MF | GO:0042169 | SH2 domain binding | 0.000333 |
| MF | GO:0005164 | tumor necrosis factor receptor binding | 0.000948 |
| MF | GO:0001614 | purinergic nucleotide receptor activity | 0.001301 |
| MF | GO:0016502 | nucleotide receptor activity | 0.001301 |
| MF | GO:0033691 | sialic acid binding | 0.001596 |
| MF | GO:0008528 | G protein-coupled peptide receptor activity | 0.001977 |
| MF | GO:0019865 | immunoglobulin binding | 0.002321 |
| MF | GO:0001653 | peptide receptor activity | 0.002383 |
| MF | GO:0045236 | CXCR chemokine receptor binding | 0.007749 |
| MF | GO:0032813 | tumor necrosis factor receptor superfamily binding | 0.009765 |
| MF | GO:0042288 | MHC class I protein binding | 0.01116 |
| MF | GO:0019864 | IgG binding | 0.019113 |
| MF | GO:0004875 | complement receptor activity | 0.024381 |
| MF | GO:0042277 | peptide binding | 0.025618 |
| MF | GO:0031406 | carboxylic acid binding | 0.034579 |
| MF | GO:0004715 | non-membrane spanning protein tyrosine kinase activity | 0.038559 |
