# Supplementary material for: Prognostic model revealing pyroptosis-related signatures in oral squamous cell carcinoma based on bioinformatics analysis
Source: Sci Rep. 2024 Mar 14;14:6149. doi: 10.1038/s41598-024-56694-y (PMC10937718; doi:10.1038/s41598-024-56694-y)
Supplement: Supplementary file 4 — Supplementary Table S4. [file 41598_2024_56694_MOESM4_ESM.docx]

**Table S4. KEGG enrichment analysis**

| **ID** | **Description** | **P adjust** |
| --- | --- | --- |
| hsa04514 | Cell adhesion molecules | 2.72E-23 |
| hsa04640 | Hematopoietic cell lineage | 1.44E-19 |
| hsa05330 | Allograft rejection | 1.44E-19 |
| hsa04060 | Cytokine-cytokine receptor interaction | 1.44E-19 |
| hsa04658 | Th1 and Th2 cell differentiation | 1.44E-19 |
| hsa04659 | Th17 cell differentiation | 7.00E-19 |
| hsa05332 | Graft-versus-host disease | 8.22E-19 |
| hsa04940 | Type I diabetes mellitus | 1.26E-18 |
| hsa04061 | Viral protein interaction with cytokine and cytokine receptor | 1.31E-17 |
| hsa04612 | Antigen processing and presentation | 9.84E-17 |
| hsa05321 | Inflammatory bowel disease | 4.62E-16 |
| hsa04672 | Intestinal immune network for IgA production | 4.66E-16 |
| hsa05320 | Autoimmune thyroid disease | 2.21E-15 |
| hsa05340 | Primary immunodeficiency | 5.49E-14 |
| hsa05150 | Staphylococcus aureus infection | 1.09E-13 |
| hsa05416 | Viral myocarditis | 6.72E-12 |
| hsa05323 | Rheumatoid arthritis | 7.30E-12 |
| hsa05140 | Leishmaniasis | 3.14E-11 |
| hsa05310 | Asthma | 3.33E-11 |
| hsa04660 | T cell receptor signaling pathway | 5.13E-11 |
| hsa04062 | Chemokine signaling pathway | 3.36E-10 |
| hsa05322 | Systemic lupus erythematosus | 7.91E-10 |
| hsa05145 | Toxoplasmosis | 1.54E-09 |
| hsa04650 | Natural killer cell mediated cytotoxicity | 2.81E-09 |
| hsa05152 | Tuberculosis | 3.05E-09 |
| hsa05164 | Influenza A | 6.90E-09 |
| hsa05166 | Human T-cell leukemia virus 1 infection | 7.24E-07 |
| hsa04145 | Phagosome | 1.12E-06 |
| hsa05235 | PD-L1 expression and PD-1 checkpoint pathway in cancer | 1.47E-06 |
| hsa05169 | Epstein-Barr virus infection | 3.00E-06 |
| hsa05143 | African trypanosomiasis | 0.000169 |
| hsa05162 | Measles | 0.00021 |
| hsa05142 | Chagas disease | 0.000223 |
| hsa04064 | NF-kappa B signaling pathway | 0.001289 |
| hsa04380 | Osteoclast differentiation | 0.00165 |
| hsa04662 | B cell receptor signaling pathway | 0.004722 |
| hsa04620 | Toll-like receptor signaling pathway | 0.005282 |
| hsa04630 | JAK-STAT signaling pathway | 0.033535 |
| hsa05144 | Malaria | 0.037204 |
