# Supplementary material for: Prognostic model revealing pyroptosis-related signatures in oral squamous cell carcinoma based on bioinformatics analysis
Source: Sci Rep. 2024 Mar 14;14:6149. doi: 10.1038/s41598-024-56694-y (PMC10937718; doi:10.1038/s41598-024-56694-y)
Supplement: Supplementary file 5 — Supplementary Table S5. [file 41598_2024_56694_MOESM5_ESM.docx]

**Table S5. GSEA analysis**

| **ID** | **NES** | **P adjust** |
| --- | --- | --- |
| KEGG_ALLOGRAFT_REJECTION | -2.40844 | 9.15E-10 |
| KEGG_ANTIGEN_PROCESSING_AND_PRESENTATION | -2.51269 | 9.15E-10 |
| KEGG_ASTHMA | -2.2556 | 9.15E-10 |
| KEGG_AUTOIMMUNE_THYROID_DISEASE | -2.43214 | 9.15E-10 |
| KEGG_CELL_ADHESION_MOLECULES_CAMS | -2.26345 | 9.15E-10 |
| KEGG_CHEMOKINE_SIGNALING_PATHWAY | -2.47176 | 9.15E-10 |
| KEGG_CYTOKINE_CYTOKINE_RECEPTOR_INTERACTION | -2.53237 | 9.15E-10 |
| KEGG_GRAFT_VERSUS_HOST_DISEASE | -2.38882 | 9.15E-10 |
| KEGG_HEMATOPOIETIC_CELL_LINEAGE | -2.38282 | 9.15E-10 |
| KEGG_INTESTINAL_IMMUNE_NETWORK_FOR_IGA_PRODUCTION | -2.35698 | 9.15E-10 |
| KEGG_JAK_STAT_SIGNALING_PATHWAY | -2.27281 | 9.15E-10 |
| KEGG_LEISHMANIA_INFECTION | -2.39942 | 9.15E-10 |
| KEGG_METABOLISM_OF_XENOBIOTICS_BY_CYTOCHROME_P450 | 2.529617 | 9.15E-10 |
| KEGG_NATURAL_KILLER_CELL_MEDIATED_CYTOTOXICITY | -2.38124 | 9.15E-10 |
| KEGG_PRIMARY_IMMUNODEFICIENCY | -2.30495 | 9.15E-10 |
| KEGG_SYSTEMIC_LUPUS_ERYTHEMATOSUS | -2.52682 | 9.15E-10 |
| KEGG_TOLL_LIKE_RECEPTOR_SIGNALING_PATHWAY | -2.2211 | 9.15E-10 |
| KEGG_TYPE_I_DIABETES_MELLITUS | -2.32943 | 9.15E-10 |
| KEGG_T_CELL_RECEPTOR_SIGNALING_PATHWAY | -2.35843 | 9.15E-10 |
| KEGG_VIRAL_MYOCARDITIS | -2.26281 | 9.15E-10 |
| KEGG_B_CELL_RECEPTOR_SIGNALING_PATHWAY | -2.05306 | 2.59E-07 |
| KEGG_DRUG_METABOLISM_CYTOCHROME_P450 | 2.296601 | 4.11E-07 |
| KEGG_FOCAL_ADHESION | -1.81744 | 4.20E-06 |
| KEGG_COMPLEMENT_AND_COAGULATION_CASCADES | -2.01763 | 8.79E-06 |
| KEGG_RETINOL_METABOLISM | 2.093895 | 2.01E-05 |
| KEGG_ECM_RECEPTOR_INTERACTION | -1.89766 | 2.46E-05 |
| KEGG_CYTOSOLIC_DNA_SENSING_PATHWAY | -1.9984 | 4.89E-05 |
| KEGG_NOD_LIKE_RECEPTOR_SIGNALING_PATHWAY | -1.92576 | 5.81E-05 |
| KEGG_STEROID_HORMONE_BIOSYNTHESIS | 2.03648 | 0.00016 |
| KEGG_FC_GAMMA_R_MEDIATED_PHAGOCYTOSIS | -1.83017 | 0.000172 |
| KEGG_FC_EPSILON_RI_SIGNALING_PATHWAY | -1.81935 | 0.000224 |
| KEGG_LYSOSOME | -1.66635 | 0.00145 |
| KEGG_LEUKOCYTE_TRANSENDOTHELIAL_MIGRATION | -1.67907 | 0.001577 |
| KEGG_ACUTE_MYELOID_LEUKEMIA | -1.79282 | 0.001669 |
| KEGG_APOPTOSIS | -1.72741 | 0.001669 |
| KEGG_SMALL_CELL_LUNG_CANCER | -1.71512 | 0.00195 |
| KEGG_PENTOSE_AND_GLUCURONATE_INTERCONVERSIONS | 1.914318 | 0.002223 |
| KEGG_PORPHYRIN_AND_CHLOROPHYLL_METABOLISM | 1.847933 | 0.003639 |
| KEGG_DRUG_METABOLISM_OTHER_ENZYMES | 1.757177 | 0.004453 |
| KEGG_RIG_I_LIKE_RECEPTOR_SIGNALING_PATHWAY | -1.75199 | 0.00547 |
| KEGG_PRION_DISEASES | -1.76398 | 0.00547 |
| KEGG_ASCORBATE_AND_ALDARATE_METABOLISM | 1.832947 | 0.008196 |
| KEGG_PROTEASOME | -1.69429 | 0.010089 |
| KEGG_OXIDATIVE_PHOSPHORYLATION | 1.444303 | 0.013541 |
| KEGG_GLUTATHIONE_METABOLISM | 1.683333 | 0.021632 |
| KEGG_PATHWAYS_IN_CANCER | -1.35369 | 0.030983 |
| KEGG_NEUROACTIVE_LIGAND_RECEPTOR_INTERACTION | -1.4227 | 0.034285 |
| KEGG_ENDOCYTOSIS | -1.39558 | 0.043602 |
| KEGG_STARCH_AND_SUCROSE_METABOLISM | 1.520002 | 0.048604 |
