# Supplementary material for: Prognostic model revealing pyroptosis-related signatures in oral squamous cell carcinoma based on bioinformatics analysis
Source: Sci Rep. 2024 Mar 14;14:6149. doi: 10.1038/s41598-024-56694-y (PMC10937718; doi:10.1038/s41598-024-56694-y)
Supplement: Supplementary file 6 — Supplementary Table S6. [file 41598_2024_56694_MOESM6_ESM.docx]

**Table S6. Univariate COX analysis and multivariate COX analysis**

**Univariate COX analysis:**

| **Univariate** | **P value** | **Hazard Ratio (95%)** |
| --- | --- | --- |
| CTLA4 | 0.00131 | 0.71 (0.576-0.875) |
| CD5 | 0.00729 | 0.771 (0.637-0.932) |
| IL12RB2 | 0.03 | 0.804 (0.66-0.979) |
| total_risk_score | 0.000286 | 5.39 (2.17-13.4) |
| sample_type_id.samples | 0.0291 | 1.05 (1-1.09) |
| shortest_dimension.samples | 1.81E-06 | 9.25 (3.71-23.1) |
| year_of_diagnosis.diagnoses | 2.58E-06 | 0.942 (0.918-0.966) |
| intermediate_dimension.samples | 0.00116 | 2.19 (1.37-3.52) |
| longest_dimension.samples | 0.000145 | 1.99 (1.39-2.83) |
| year_of_form_completion | 0.00409 | 0.795 (0.68-0.93) |
| year_of_initial_pathologic_diagnosis | 2.58E-06 | 0.942 (0.918-0.966) |
| program | 0.000575 | 3.55 (1.73-7.32) |
| age_at_index.demographic | 0.00679 | 1.02 (1-1.03) |
| days_to_birth.demographic | 0.00712 | 1 (1-1) |
| days_to_death.demographic | 2.73E-53 | 0.846 (0.829-0.865) |
| year_of_birth.demographic | 1.12E-05 | 0.975 (0.963-0.986) |
| age_at_diagnosis.diagnoses | 0.00712 | 1 (1-1) |
| days_to_last_follow_up.diagnoses | 2.45E-26 | 0.992 (0.99-0.993) |
| additional_pharmaceutical_therapy | | |
| NO | 0.00064 | 1.72 (0.951-3.1) |
| YES | 0.00064 | 3.19 (1.67-6.09) |
| additional_radiation_therapy | | |
| NO | 5.75E-05 | 1.38 (0.705-2.72) |
| YES | 5.75E-05 | 3.61 (2.03-6.42) |
| additional_surgery_locoregional_procedure | | |
| NO | 0.000381 | 2.62 (1.51-4.55) |
| YES | 0.000381 | 2.55 (1.12-5.79) |
| additional_surgery_metastatic_procedure | | |
| NO | 0.0022 | 2.68 (1.54-4.64) |
| age_at_initial_pathologic_diagnosis | 0.00679 | 1.02 (1-1.03) |
| days_to_new_tumor_event_after_initial_treatment | 0.000548 | 0.994 (0.99-0.997) |
| disease_after_curative_tx | | |
| NO | 2.25E-08 | 0.225 (0.134-0.377) |
| YES | 2.25E-08 | 1.55 (0.811-2.94) |
| followup_case_report_form_submission_reason | | |
| Additional New Tumor Event | 9.57E-07 | 1.34 (0.665-2.69) |
| Scheduled Follow-up Submission | 9.57E-07 | 0.466 (0.341-0.637) |
| followup_treatment_success | | |
| Complete Remission/Response | 1.91E-14 | 0.259 (0.173-0.387) |
| Partial Remission/Response | 1.91E-14 | 1.97 (0.481-8.04) |
| Persistent Disease | 1.91E-14 | 1.81 (0.875-3.75) |
| Progressive Disease | 1.91E-14 | 1.92 (1.29-2.87) |
| Stable Disease | 1.91E-14 | 0.733 (0.18-2.99) |
| laterality |  |  |
| Left | 0.000455 | 0.727 (0.503-1.05) |
| Midline | 0.000455 | 0.896 (0.464-1.73) |
| Right | 0.000455 | 0.44 (0.3-0.646) |
| lost_follow_up | |  |
| NO | 6.32E-08 | 0.427 (0.316-0.577) |
| YES | 6.32E-08 | 0.229 (0.0564-0.93) |
| lymphovascular_invasion_present | | |
| NO | 0.0449 | 0.842 (0.589-1.2) |
| YES | 0.0449 | 1.35 (0.921-1.99) |
| method_of_curative_tx | | |
| Chemotherapy (not given concurrently) | 7.88E-05 | 1.5 (0.209-10.8) |
| Concurrent Chemotherapy | 7.88E-05 | 0.741 (0.4-1.37) |
| Radiation | 7.88E-05 | 0.349 (0.143-0.854) |
| Surgery | 7.88E-05 | 0.281 (0.159-0.497) |
| new_neoplasm_event_occurrence_anatomic_site | | |
| Cervical Lymph Nodes | 0.000298 | 3.33 (1.46-7.56) |
| Distant Metastasis | 0.000298 | 2.62 (1.16-5.96) |
| Oral Cavity | 0.000298 | 2.79 (1.3-6) |
| Oropharynx | 0.000298 | 3.65 (0.9-14.8) |
| new_tumor_event_after_initial_treatment | | |
| NO | 2.61E-05 | 0.794 (0.439-1.44) |
| YES | 2.61E-05 | 2.87 (1.8-4.59) |
| number_of_lymphnodes_positive_by_he | 5.66E-06 | 1.07 (1.04-1.1) |
| pathologic_N | |  |
| N0 | 2.34E-05 | 0.949 (0.402-2.24) |
| N1 | 2.34E-05 | 0.783 (0.305-2.02) |
| N2 | 2.34E-05 | 1.66 (0.5-5.5) |
| N2a | 2.34E-05 | 2.27 (0.453-11.3) |
| N2b | 2.34E-05 | 1.82 (0.764-4.33) |
| N2c | 2.34E-05 | 3.46 (1.38-8.71) |
| N3 | 2.34E-05 | 3.59 (0.718-18) |
| NX | 2.34E-05 | 1.54 (0.639-3.71) |
| pathologic_T | |  |
| T1 | 0.000376 | 0.658 (0.19-2.28) |
| T2 | 0.000376 | 1.28 (0.453-3.6) |
| T3 | 0.000376 | 2.67 (0.939-7.59) |
| T4 | 0.000376 | 3.17 (0.907-11.1) |
| T4a | 0.000376 | 2.54 (0.898-7.18) |
| T4b | 0.000376 | 2.86 (0.514-15.9) |
| TX | 0.000376 | 1.93 (0.533-7) |
| patient_death_reason | | |
| Other, non-malignant disease | 2.36E-07 | 2.89 (1.28-6.57) |
| Related to Head & Neck Cancer | 2.36E-07 | 4.25 (2.43-7.43) |
| perineural_invasion_present | | |
| NO | 0.00804 | 0.701 (0.46-1.07) |
| YES | 0.00804 | 1.27 (0.896-1.81) |
| person_neoplasm_cancer_status | | |
| TUMOR FREE | 3.59E-23 | 0.205 (0.138-0.305) |
| WITH TUMOR | 3.59E-23 | 1.6 (1.13-2.26) |
| postoperative_rx_tx | |  |
| NO | 0.000394 | 0.567 (0.406-0.79) |
| YES | 0.000394 | 0.453 (0.286-0.717) |
| presence_of_pathological_nodal_extracapsular_spread | | |
| Gross Extension | 2.66E-08 | 2.4 (1.35-4.24) |
| Microscopic Extension | 2.66E-08 | 2.22 (1.43-3.43) |
| No Extranodal Extension | 2.66E-08 | 0.714 (0.501-1.02) |
| primary_lymph_node_presentation_assessment | | |
| NO | 0.000868 | 2.41 (1.23-4.72) |
| YES | 0.000868 | 0.958 (0.572-1.6) |
| primary_therapy_outcome_success | | |
| Complete Remission/Response | 8.78E-15 | 0.391 (0.283-0.54) |
| Partial Remission/Response | 8.78E-15 | 2.8 (1.01-7.78) |
| Persistent Disease | 8.78E-15 | 1.54 (0.56-4.22) |
| Progressive Disease | 8.78E-15 | 2.87 (1.7-4.86) |
| Stable Disease | 8.78E-15 | 0.591 (0.0818-4.26) |
| radiation_therapy | |  |
| NO | 1.20E-06 | 0.74 (0.516-1.06) |
| YES | 1.20E-06 | 0.381 (0.263-0.553) |
| smokeless_tobacco_use_at_diag | | |
| NO | 0.0162 | 0.638 (0.469-0.869) |
| YES | 0.0162 | 1.02 (0.324-3.22) |
| tumor_stage.diagnoses | | |
| stage i | 0.000732 | 0.386 (0.127-1.17) |
| stage ii | 0.000732 | 0.751 (0.397-1.42) |
| stage iii | 0.000732 | 1.04 (0.553-1.95) |
| stage iva | 0.000732 | 1.63 (0.931-2.84) |
| stage ivb | 0.000732 | 2.33 (0.768-7.1) |
| sample_type | |  |
| Primary Tumor | 0.00121 | 0.0422 (0.00558-0.319) |
| Solid Tissue Normal | 0.00121 | 0.0668 (0.00855-0.522) |

**Multivariate COX analysis:**

| **Multivariate** | **P value** | **Hazard Ratio (95%)** |
| --- | --- | --- |
| CTLA4 | 0 | 2.3e+116 (4.6e+115-1.1e+117) |
| CD5 | 0 | 8.8e+60 (2.5e+60-3.2e+61) |
| IL12RB2 | 0 | 7.3e+76 (2.3e+76-2.3e+77) |
| total_risk_score | 0 | Inf (Inf-Inf) |
| age_at_initial_pathologic_diagnosis | 9.50E-34 | 0.37 (0.31-0.43) |
| days_to_new_tumor_event_after_initial_treatment | 0.0054 | 0.77 (0.64-0.93) |
| additional_pharmaceutical_therapy | | |
| NO | 0.96 | 1.2e-55 (0-Inf) |
| YES | 0.94 | 1.1e-78 (0-Inf) |
| additional_radiation_therapy | | |
| NO | 0.98 | 1.6e+25 (0-Inf) |
| YES | 1 | 1 (0-Inf) |
| additional_surgery_locoregional_procedure | | |
| NO | 0.94 | 5.3e-93 (0-Inf) |
| YES | 0.97 | 3.6e+61 (0-Inf) |
| additional_surgery_metastatic_procedure | | |
| NO | 0.95 | 1e+67 (0-Inf) |
| YES | 1 | 3.3e+29 (0-Inf) |
| followup_case_report_form_submission_reason | | |
| Additional New Tumor Event | 0.98 | 1.1e+69 (0-Inf) |
| Scheduled Follow-up Submission | 0.97 | 3.9e-38 (0-Inf) |
| followup_treatment_success | | |
| Complete Remission/Response | 0.92 | 3.7e-179 (0-Inf) |
| Persistent Disease | 0.94 | 1.1e-65 (0-Inf) |
| Progressive Disease | 1 | 1.4e+07 (0-Inf) |
| pathologic_T | |  |
| T2 | 0 | 3.4e+141 (2.1e+140-5.4e+142) |
| T3 | 0 | 1e+141 (6.5e+139-1.6e+142) |
| T4a | 0.96 | 1.6e+91 (0-Inf) |
| T4b | 0.93 | 3e+154 (0-Inf) |
| TX | 0.97 | 2.2e+55 (0-Inf) |
| perineural_invasion_present | | |
| NO | 1 | 1 (0-Inf) |
| YES | 1 | 1 (0.063-16) |
| person_neoplasm_cancer_status | | |
| TUMOR FREE | 1 | 1 (0.063-16) |
| WITH TUMOR | 1 | 1 (0-Inf) |
